# Electron spin dynamics during microwave pulses studied by 94 GHz chirp and phase-modulated EPR experiments

**DOI:** 10.5194/mr-6-43-2025

**Published:** 2025-02-19

**Authors:** Marvin Lenjer, Nino Wili, Fabian Hecker, Marina Bennati

**Affiliations:** 1 RG EPR Spectroscopy, Max Planck Institute for Multidisciplinary Sciences, Am Fassberg 11, 37077 Göttingen, Germany; 2 Institute for Physical Chemistry, Georg August University Göttingen, Tammanstrasse 6, 37077 Göttingen, Germany; 3 Interdisciplinary Nanoscience Center, Aarhus University, Gustav Wieds Vej 14, 8000 Aarhus C, Denmark; 4 Center for Hyperpolarization in Magnetic Resonance, Danish Technical University, Oerstedsplads 349, 2800 Kongens Lyngby, Denmark

## Abstract

Electron spin dynamics during microwave irradiation are of increasing interest in electron paramagnetic resonance (EPR) spectroscopy, as locking electron spins into a *dressed* state finds applications in EPR and dynamic nuclear polarization (DNP) experiments. Here, we show that these dynamics can be probed by modern pulsed EPR experiments that use arbitrary waveform generators to produce shaped microwave pulses. We employ phase-modulated pulses to measure Rabi nutations, echoes, and echo decays during spin locking of a BDPA (1,3-bisdiphenylene-2-phenylallyl) radical at 94 GHz EPR frequency. Depending on the initial state of magnetization, different types of echoes are observed. We analyze these distinct coherence transfer pathways and measure the decoherence time $T_{2\rho }$ , which is a factor of 2–3 times longer than $T_{m}$ . Furthermore, we use chirped Fourier transform EPR to detect the evolution of magnetization profiles. Our experimental results are well reproduced using a simple density matrix model that accounts for $T_{2\rho }$ relaxation in the spin lock (tilted) frame. The results provide a starting point for optimizing EPR experiments based on hole burning, such as electron–nuclear double resonance or ELectron–electron DOuble Resonance (ELDOR)-detected NMR.

## 1 Introduction

Long microwave (MW) pulses with a duration of several microseconds are building blocks for pulse sequences in electron paramagnetic resonance (EPR) spectroscopy. They serve as preparation pulses to drive forbidden EPR transitions in hyperfine spectroscopy experiments like ELectron–electron DOuble Resonance (ELDOR)-detected nuclear magnetic resonance (EDNMR) [Bibr bib1.bibx55] and can also lock spins into a *dressed* state, characterized by a distinct reorganization of their energy states [Bibr bib1.bibx12]. This enables electron–nuclear polarization transfer via cross-polarization (CP) under modified Hartmann–Hahn matching conditions as NOVEL or eNCP [Bibr bib1.bibx28]. Spin locking (SL) can also be harnessed to decouple spins from their surroundings, resulting in longer decoherence times of electron spins in EPR and optically detected magnetic resonance due to decoupling of nuclear interactions [Bibr bib1.bibx35]. SL experiments were recently proposed for electron–electron distance determination, where the upper limit of detectable distances is determined by the electron decoherence time [Bibr bib1.bibx67]. The altered energy landscape of electron spins locked by a MW field and under relaxation results in distinct spin dynamics, which are not yet fully explored.

**Figure 1 Ch1.F1:**
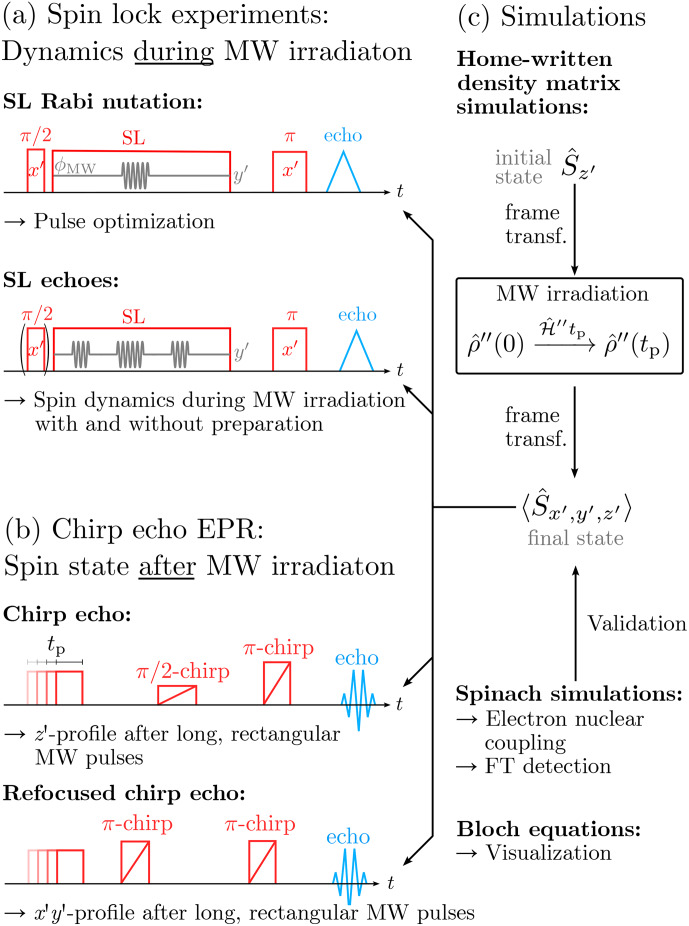
Overview of the pulse sequences applied throughout this work and connection to the computational approach. While the spin lock experiments **(a)** were used as an analytical tool to monitor spin dynamics during MW irradiation, chirp echo EPR provided insights into the spin state following long, rectangular MW pulses. The results were analyzed using density matrix simulations. Additional calculations based on the Spinach library [Bibr bib1.bibx29] and the Bloch equations [Bibr bib1.bibx6] were used for validation and visualization **(c)**.

One important aspect is the effect of decoherence during spin locking (i.e., $T_{2\rho }$ relaxation), which has recently been discussed in the context of EPR spectroscopy by [Bibr bib1.bibx67]. Some examples of treating this process exist in the literature. Most of these approaches rely on a phenomenological description of decoherence by rate equations but differ in the chosen interaction frame, which in turn determines the spin states that are affected by relaxation. For example, in a theoretical treatment of CP-ENDOR [Bibr bib1.bibx5], we approximated the final state after a long SL pulse by propagating the spin density matrix in the dressed state yet without relaxation terms, followed by a heuristic implementation of $T_{2\rho }$ that eliminates off-diagonal matrix elements. [Bibr bib1.bibx30] described relaxation during MW irradiation in dynamic nuclear polarization experiments using density matrix propagation in the eigenframe of the equilibrium Hamiltonian without MW irradiation. Similarly, [Bibr bib1.bibx27] and [Bibr bib1.bibx13] proposed to model the effect of NMR shaped pulses or EDNMR preparation pulses with the Bloch equations in the rotating frame, including $T_{m}$ relaxation. In contrast, [Bibr bib1.bibx15], [Bibr bib1.bibx14], and [Bibr bib1.bibx45] expressed relaxation rates during irradiation in the eigenframe of the complete Hamiltonian including an irradiation term (i.e., the tilted frame) when analyzing the effect of dipolar interaction, molecular motion, and chemical exchange to the overall coherence decay rate. Very recently, [Bibr bib1.bibx37] calculated electron $T_{2\rho }$ decays not using rate equations but from both the analytical pair product approximation and cluster correlation expansion. Their results show that a rigorous, quantitative treatment of decoherence during SL is currently challenging. Therefore, phenomenological treatment of relaxation using rate equations is still highly relevant to describe SL experiments.

This paper presents our efforts to gain insight into the electron spin dynamics of long MW pulses ( $T_{2\rho }≲t_{\mathrm{pulse}}≲T_{1\rho }$ ). We focused on rationalizing decoherence effects and, ultimately, the trajectories of spins during MW irradiation to determine correct excitation profiles in EPR hole-burning experiments. Simultaneously, the experiments showcase the applicability of shaped pulses at our commercial W-band spectrometer. In the literature, most shaped-pulse experiments were performed at MW frequencies lower or equal to Q-band frequencies (34 GHz ) [Bibr bib1.bibx21]. The use of shaped MW pulses at high frequencies has been so far constrained to inversion pulses [Bibr bib1.bibx2] or highly specialized experimental setups [Bibr bib1.bibx38].

Experimentally, we followed two main routes. On the one hand, we performed pulse experiments during SL by coherently manipulating the locked spins using periodic phase modulations (PM) of the locking field [Bibr bib1.bibx25]. We report 94GHz SL Rabi nutations, echoes, and echo decays measured with pulse sequences established by [Bibr bib1.bibx67] that were analyzed with electron spin dynamic simulations (Fig. [Fig Ch1.F1]a). On the other hand, we applied chirp echo Fourier transform (FT) EPR spectroscopy (CHEESY) [Bibr bib1.bibx66] to measure the excitation profiles of long, rectangular MW pulses in the regime between Rabi nutation behavior and steady-state conditions (Fig. [Fig Ch1.F1]b). While the first class of experiments allows for observation of the dynamics during MW irradiation, the second class enables observation of the spin state immediately after a MW pulse. Therefore, the two approaches provide complementary information that we can use as a basis for density matrix simulations of an isolated electron spin under MW irradiation. Using the $T_{2\rho }$ values obtained from the PM experiments, we were able to simulate the CHEESY excitation profiles. Comparison with simulations using the well-established Spinach library [Bibr bib1.bibx29] showed consistent results when using the suited interaction frame. Together with calculations based on the Bloch equations, we demonstrate that, for MW irradiation periods on the order of $T_{2\rho }$ , calculating in the correct frame of reference is crucial for describing the experimental data.

## 2 Theoretical description

In this section, we provide the spin Hamiltonians used for the simulations of PM experiments and chirp echo profiles in their respective interaction frames. As will become evident from the results, a theoretical description based on the simple model of an isolated S=1/2 electron spin under MW irradiation, including a MW frequency offset, is sufficient to reproduce our experimental results. In the following, we refer to a spin system during free evolution as the *bare* state in contrast to spins subject to continuous MW irradiation, which are in the *dressed* or SL state. We label laboratory frame operators without a prime, rotating frame operators with a single prime (^′^), and tilted frame (eigenframe during microwave irradiation) operators with a double prime (^′′^). Operators in the nutating frame (interaction frame for PM pulses during spin lock) are denoted with an asterisk (^*^). Furthermore, we indicate how the time evolution of the density matrix in the different frames can be computed under the effect of decoherence. All equations are expressed in angular frequency units ( ω ), while experimental results are reported in frequency units ( ν=ω/2π ) as recorded.

### 2.1 Spin Hamiltonian

The laboratory frame Hamiltonian $\hat{H}$ for an isolated electron spin under irradiation with linearly polarized MW radiation in the x direction contains the electron Zeeman and the microwave irradiation terms:

1 $$\hat{H}(t)=\omega_{0}{\hat{S}}_{z}+2\omega_{1}\mathrm{cos}(\omega_{\mathrm{MW}}t+\phi_{\mathrm{MW}}){\hat{S}}_{x},$$

where $\omega_{0}$ is the Larmor frequency; $\omega_{\mathrm{MW}}$ and $\phi_{\mathrm{MW}}$ are the MW frequency and phase, respectively; and $\omega_{1}$ is the Rabi frequency proportional to the MW $B_{1}$ field. To eliminate the time dependence, $\hat{H}(t)$ is transformed into a rotating frame with ${\hat{U}}_{1}(t)=\mathrm{exp}(-i\omega_{\mathrm{MW}}t{\hat{S}}_{z})$ [Bibr bib1.bibx1]. Subsequent application of the rotating-wave approximation (RWA) yields the time-independent rotating frame Hamiltonian:

2H^′=U^1†(t)H^(t)U^1(t)-iU^1†(t)dU^1(t)dt3≈RWAΩSS^z′+ω1cos⁡ϕMWS^x′+ω1sin⁡ϕMWS^y′,

where $\Omega_{S}=\omega_{0}-\omega_{\mathrm{MW}}$ is the frequency offset of the MW to the electron Larmor frequency. Unless stated otherwise, MW frequency irradiation is assumed to be applied along $y^{\prime }$ (i.e., $\phi_{\mathrm{MW}}=90{}^{\circ}$ ), which is consistent with our experimental SL field.

#### 2.1.1 PM pulses and nutating frame

In the rotating frame, the Hamiltonian ${\hat{H}}_{\mathrm{PM}}^{\prime }$ during a PM pulse is defined as in Eq. (4), including a periodic variation of the phase $\phi_{\mathrm{MW}}(t)$ (Eq. 5) that is matched with $\omega_{1}$ [Bibr bib1.bibx67].

4H^PM′=ΩSS^z′+ω1cos⁡ϕMW(t)S^x′+ω1sin⁡ϕMW(t)S^y′,5ϕMW(t)=ϕ0+aPMcos⁡ωPMt+ϕPM,

where $\phi_{0}$ is the spin lock phase, $a_{\mathrm{PM}}$ the modulation depth of the phase modulation, and $\omega_{\mathrm{PM}}$ its frequency. $\phi_{\mathrm{PM}}$ is the phase of what will later be defined as the PM pulse. Assuming a small modulation amplitude $a_{\mathrm{PM}}$ and strong driving conditions (i.e., $\omega_{1}\gg \Omega_{S}$ ), this expression can be transformed into the nutating frame by a rotation with ${\hat{U}}_{\mathrm{PM}}(t)=\mathrm{exp}\left(-i\omega_{\mathrm{PM}}t{\hat{S}}_{y^{\prime }}\right)$ , yielding Eq. ([Disp-formula Ch1.E6]) after application of the rotating wave approximation [Bibr bib1.bibx25].

6 $${\hat{H}}_{\mathrm{PM}}^{*}\overset{\mathrm{RWA}}{\approx }\Omega_{d}{\hat{S}}_{y^{*}}+\frac{\omega_{1}a_{\mathrm{PM}}}{2}\left(-\mathrm{cos}\left(\phi_{\mathrm{PM}}\right){\hat{S}}_{x^{*}}+\mathrm{sin}\left(\phi_{\mathrm{PM}}\right){\hat{S}}_{z^{*}}\right)$$

Here, $\Omega_{d}=\omega_{1}-\omega_{\mathrm{PM}}$ is a nutating frame offset that is unavoidable due to MW inhomogeneity. In contrast to the experiments performed by [Bibr bib1.bibx67], our experiments do not fulfill the abovementioned strong driving condition. Therefore, we used Eq. (4) for numerical simulations. Nevertheless, Eq. (6) provides an effective way of understanding PM experiments, demonstrating the analogy to a rotating frame experiment (see Eq. 3) with pulses along $x^{*}$ or $z^{*}$ and with a nutation frequency of $\frac{\omega_{1}a_{\mathrm{PM}}}{2}$ . The validity of Eq. ([Disp-formula Ch1.E6]) under our experimental conditions is discussed in the Results section (see Sect. [Sec Ch1.S4.SS1.SSS1]).

#### 2.1.2 Tilted frame

During constant MW irradiation, a spin ensemble in the rotating frame is subject to an effective field $\omega_{\mathrm{eff}}=\Omega_{S}z^{\prime }+\omega_{1}y^{\prime }$ described by the vector sum of the spin offsets and the microwave field. Therefore, ${\hat{H}}^{\prime }$ is not diagonal. Diagonalization of ${\hat{H}}^{\prime }$ leads to a rearrangement of the energy states due to the altered quantization axis [Bibr bib1.bibx52] and is achieved by a rotation of ${\hat{H}}^{\prime }$ with ${\hat{U}}_{2}$ around $x^{\prime }$ into a so-called tilted or spin-locked frame (Eq. 7). Note that, in this case, we apply the rotations on the operator and not the coordinate frame so that ${\hat{H}}^{\prime \prime }(t)$ is obtained by Eq. (8) [Bibr bib1.bibx22].

7U^2=exp⁡-iθS^x′,8H^′′(t)=U^2H^′(t)U^2†.

In Eq. (7), $\theta ={\mathrm{Tan}}^{-1}(\omega_{1}/\Omega_{S})$ is the tilt angle of the $z^{\prime \prime }$ axis in the Hamiltonian eigenframe with respect to $z^{\prime }$ , defined such that θ∈[0°,180°] for $\omega_{1}>0$ . ${\mathrm{Tan}}^{-1}()$ is the four-quadrant inverse tangent (e.g., implemented in MATLAB as the atan2() command) that returns the correct quadrant of θ by using $\omega_{1}$ and $\Omega_{S}$ as separate input values [Bibr bib1.bibx5]. The transformation yields the tilted-frame Hamiltonian ${\hat{H}}^{\prime \prime }$ where $z^{\prime \prime }$ is aligned with $\omega_{\mathrm{eff}}$ (Eq. 9).

9 $${\hat{H}}^{\prime \prime }=\omega_{\mathrm{eff}}{\hat{S}}_{z^{\prime \prime }}$$

This transformation is necessary to implement spin relaxation as discussed in the following.

### 2.2 Density operator and time evolution

The spin system evolution can be calculated using the density operator formalism. Here, the Hamiltonian ( $\hat{H}$ , ${\hat{H}}^{\prime }$ , ${\hat{H}}^{\prime \prime }$ , or ${\hat{H}}^{*}$ ) acts on an ensemble of electron spins described by a time-dependent 2×2 density operator $\hat{\rho }(t)$ in the same frame of reference (i.e., $\hat{\rho }(t)$ , ${\hat{\rho }}^{\prime }(t)$ , ${\hat{\rho }}^{\prime \prime }(t)$ , or ${\hat{\rho }}^{*}(t)$ ). For the frame transformation of the density matrix, the previously defined transformation matrices are used with the same sense of rotation as for the corresponding Hamiltonian [Bibr bib1.bibx5] (e.g., ${\hat{\rho }}^{\prime \prime }(t)={\hat{U}}_{2}{\hat{\rho }}^{\prime }(t){\hat{U}}_{2}^{†}$ ). The time evolution of any $\hat{\rho }$ is computed from the solution of the Liouville–von Neumann equation in the respective frame and under assumption of time-independent Hamilton operators; for example, in the rotating frame,

10 $${\hat{\rho }}^{\prime }(t)={\hat{U}}^{\prime }(t){\hat{\rho }}^{\prime }(0){{\hat{U}}^{\prime }}^{†}(t),$$

where ${\hat{U}}^{\prime }(t)=\mathrm{exp}(-i{\hat{H}}^{\prime }t)$ is a time-dependent propagator, and ${\hat{\rho }}^{\prime }(0)$ is the initial density operator. To include relaxation into the time evolution of ${\hat{\rho }}^{\prime }$ , calculations are performed in Liouville space [Bibr bib1.bibx30]. For this purpose, the Hamiltonian in Hilbert space is converted to the corresponding Liouvillian superoperator $\hat{\hat{L}}$ as shown in Eq. ([Disp-formula Ch1.E11]) for ${\hat{H}}^{\prime }$ . The uppercase T denotes the transposed matrix and, 1 is the 2×2 unit matrix [Bibr bib1.bibx40].

11 $${\hat{\hat{L}}}^{\prime }=1⊗{\hat{H}}^{\prime }-{{\hat{H}}^{\prime }}^{T}⊗1$$

The respective density operator in Hilbert space is converted into a four-element density vector operator $\hat{\rho }(t)$ . The ordering of the matrix elements into a vector is shown in Eq. ([Disp-formula Ch1.E12]) where α and β are spin eigenstates [Bibr bib1.bibx23].

12 $${\hat{\rho }}^{\prime }(t)=\left(\begin{array}{c} {\hat{\rho }}_{\alpha \alpha }^{\prime }(t)\\ {\hat{\rho }}_{\alpha \beta }^{\prime }(t)\\ {\hat{\rho }}_{\beta \alpha }^{\prime }(t)\\ {\hat{\rho }}_{\beta \beta }^{\prime }(t) \end{array}\right)$$

The evolution of the density vector operator is then evaluated using a propagation superoperator ${\hat{\hat{U}}}^{\prime }(t)$ and the solution of the Liouville-von Neumann equation in Liouville space:

13 $${\hat{\rho }}^{\prime }(t)={\hat{\hat{U}}}^{\prime }(t){\hat{\rho }}^{\prime }(0)=\mathrm{exp}\left(-i{\hat{\hat{L}}}^{\prime }t\right){\hat{\rho }}^{\prime }(0).$$

Starting from a Boltzmann-populated equilibrium density operator $\hat{\rho }(0)={\hat{\rho }}^{\prime }(0)\propto {\hat{S}}_{z^{\prime }}$ , Eq. ([Disp-formula Ch1.E13]) allows for numerical calculation of electron spin ensemble evolution under MW irradiation using matrix exponentials with standard mathematics software such as MATLAB. For better readability, we define the initial density matrix as positive (i.e., ${\hat{\rho }}^{\prime }(0)={\hat{S}}_{z^{\prime }}$ ) so that the sign of the quantum mechanical expectation values is the same as of corresponding macroscopic magnetization vectors (i.e., $M(0)=(0,0,M_{0}{)}^{T}$ ). Note that, due to the negative gyromagnetic ratio of the electron, the equilibrium density matrix in the high-temperature approximation is ${\hat{\rho }}^{\prime }(0)=1/2-|R|{\hat{S}}_{z^{\prime }}$ , where R is a thermodynamic population factor [Bibr bib1.bibx5]. 1/2 does not evolve and is neglected in the following. In the tilted frame, the equilibrium density matrix transforms to ${\hat{\rho }}^{\prime \prime }(0)={\hat{U}}_{2}{\hat{\rho }}^{\prime }(0){\hat{U}}_{2}^{†}=\mathrm{cos}(\theta ){\hat{S}}_{z^{\prime \prime }}-\mathrm{sin}(\theta ){\hat{S}}_{y^{\prime \prime }}$ .

To describe relaxation *during* MW irradiation, relaxation terms are added in the tilted frame and, thus, acting during propagation of the ${\hat{\rho }}^{\prime \prime }(t)$ density matrix. For this, a relaxation superoperator is defined using a phenomenological decoherence rate [Bibr bib1.bibx24]. In our simulations, we consider only transverse relaxation since the effect of longitudinal relaxation was found to be almost negligible on the timescale of our experiments. As relaxation occurs in the tilted frame, we name it $T_{2\rho }$ in agreement with the literature [Bibr bib1.bibx15], and the corresponding relaxation superoperator is ${\hat{\hat{R}}}_{2\rho }$ .

14 $${\hat{\hat{R}}}_{2\rho }=\left(\begin{array}{cccc} 0 & & & \\ & -\frac{1}{T_{2\rho }} & & \\ & & -\frac{1}{T_{2\rho }} & \\ & & & 0 \end{array}\right)$$

Evolution of the spin system can then be calculated by solving the Liouville-von Neumann equation (Eq. [Disp-formula Ch1.E13]) with a modified propagation superoperator ${\hat{\hat{U}}}^{\prime \prime }(t)$ that includes ${\hat{\hat{R}}}_{2\rho }$ [Bibr bib1.bibx24].

15 $${\hat{\hat{U}}}^{\prime \prime }(t)=\mathrm{exp}\left(\left(-i{\hat{\hat{L}}}^{\prime \prime }+{\hat{\hat{R}}}_{2\rho }\right)t\right)$$

Note that the effect of ${\hat{\hat{R}}}_{2\rho }$ in Eq. ([Disp-formula Ch1.E15]) is the exponential dampening of the coherent terms ${\hat{\rho }}_{\alpha \beta }^{\prime \prime }(t)$ and ${\hat{\rho }}_{\beta \alpha }^{\prime \prime }(t)$ in the density vector operator ${\hat{\rho }}^{\prime \prime }(t)$ of Eq. ([Disp-formula Ch1.E12]). Only if the density operator is in the eigenbasis of the full spin Hamiltonian will these entries correspond to pure coherence. Otherwise, they correspond to mixtures of populations and coherences, which changes the relaxation behavior (vide infra).

## 3 Materials and methods

### 3.1 EPR sample

A standard powder sample of 0.1% ( m/m ) 1,3-bisdiphenylene-2-phenylallyl (BDPA) in a polystyrene matrix was used for EPR experiments throughout this work. At W-band frequencies, the powder spectrum of this radical features a single Gaussian line with a full width at half maximum (FWHM) of approximately 8.8G or 24MHz , due to unresolved hyperfine couplings below 10MHz (see Fig. [Fig App1.Ch1.S1.F15]b). Measurements were done at temperatures between 50 and 100K .

### 3.2 Spectrometer configuration and characterization

All experiments were performed on a commercial Bruker E680 W-band EPR spectrometer equipped with an ENDOR resonator (model EN600-1021H) and a liquid helium cryostat (Oxford Instruments). Pulse sequences were set up with the pulse programming language PulseSPEL implemented in Bruker's Xepr operating software. Shaped pulses were generated using a Bruker SpinJet arbitrary waveform generator (AWG) with a 1.6GS s^−1^ (where GS represents gigasamples) sampling rate and ±400MHz bandwidth. In our setup, W-band frequency is reached via a three-step, heterodyne mixing scheme. First, a constant frequency $\nu_{x}$ in the range of 9.6±0.4GHz is generated by an X-band source. Second, this carrier wave is mixed with the two AWG output channels I(t) and Q(t) for the real and imaginary parts, respectively, using an IQ mixer. Third, this shaped X-band pulse is mixed with the output of a phase-locked oscillator at $\nu_{\mathrm{PLO}}=84.5\;\mathrm{GHz}$ to reach the pulse shape at W-band frequencies (see Sect. [Sec App1.Ch1.S1.SS1]). As the AWG frequency is centered at zero, active compensation of the input amplitude imbalance and filtering are applied to minimize the contribution of $\nu_{\mathrm{LO}}=\nu_{x}+\nu_{\mathrm{PLO}}$ (“LO leakage”) and mirror frequencies. The pulses are amplified with a solid-state power amplifier (SSPA, Quinstar) with 2 W output power and transferred to the resonator through a circulator. Spin echoes are recorded after demodulation with $\nu_{\mathrm{PLO}}$ and subsequent amplification via a low-noise amplifier. Quadrature detection is done using $\nu_{x}$ as a continuous reference. To characterize our experimental conditions, we analyzed the phase stability of our setup following [Bibr bib1.bibx21]. The experiment (see Sect. [Sec App1.Ch1.S1.SS2]) showed that the data suffer from phase noise that can, however, be averaged out using multiple scans. We acquired the MW resonator profile in a range of 600MHz using standard Rabi nutation experiments measured at different local oscillator frequencies $\nu_{\mathrm{LO}}$ (see Fig. [Fig App1.Ch1.S1.F11]a). At 50K , we obtained a representative resonator bandwidth of approximately 140MHz and a maximal Rabi frequency of $\nu_{1}\approx 15\;\mathrm{MHz}$ . Both values depend on the tuning conditions. Likewise, the amplification curve of the SSPA was measured with Rabi nutation experiments for the whole range of input power levels. This experiment showed clear nonlinearity of the MW amplification that was compensated for during pulse shape generation (see Fig. [Fig App1.Ch1.S1.F11]b).

### 3.3 Experiments with phase-modulated pulses

If not denoted otherwise, all phase-modulated (PM) experiments during SL were initiated by a rectangular π/2 pulse immediately followed by a 90° phase-shifted SL pulse. During this SL, the PM pulses were applied (see Fig. [Sec App1.Ch1.S1.SS4]). The locked spins were read out by a rectangular π pulse in the rotating frame, after a delay τ , which led to a spin echo at 2τ after the SL pulse. Integration of this echo yielded the detected signal.

For each data point of a parameter sweep (e.g., $t_{\mathrm{PM}}$ or $\tau_{2}$ , vide infra) during a PM experiment, a pulse shape file ( I/Q values), including the spin lock as well as all PM pulses, was generated with a home-written MATLAB script, indexed, and uploaded to the AWG. The complete experiment, including rectangular detection pulses and delays, was programmed in PulseSPEL. Analogously, PM pulse phase cycling (PC) was achieved by generating individual pulse shapes for each combination of PM phases. Due to the memory limitations of the SpinJet AWG, the pulse shape files were generated with 10ns time resolution of individual I/Q values, which was well below the AWG sampling rate but sufficient to encode the PM pulses for the maximal modulation frequency with only minimal distortions of the pulse shape (see Fig. [Sec App1.Ch1.S1.SS4]). PM pulses were optimized with the experimental procedure reported by [Bibr bib1.bibx67]. The modulation frequency $\omega_{\mathrm{PM}}$ was calibrated by monitoring the effect of a long PM pulse with low modulation depth $a_{\text{PM}}=0.04$ on the intensity of the final spin echo as a function of $\omega_{\mathrm{PM}}$ . The PM frequency with the strongest effect was chosen for the following experiments as this corresponds to the matching condition $\omega_{\mathrm{PM}}=\omega_{1}$ (see Sect. [Sec App1.Ch1.S1.SS5]). For the SL echoes, $a_{\mathrm{PM}}=0.4$ was used, and the PM pulse lengths were determined from the SL Rabi nutations.

### 3.4 Generation of chirp pulses

The chirp echo EPR experiments were set up based on the work by [Bibr bib1.bibx17] and [Bibr bib1.bibx66]. Additional information can be found in the work by [Bibr bib1.bibx21] and [Bibr bib1.bibx58]. Chirp pulses with a frequency range of 200 MHz and lengths of 1µs and 0.5µs were generated with a home-written MATLAB routine. The pulse shapes featured a nonlinear frequency sweep as a compensation for the previously measured resonator profile. This ensured approximately constant critical adiabaticity $Q_{\mathrm{crit}}$ during the frequency sweep [Bibr bib1.bibx3] (see Sect. [Sec App1.Ch1.S1.SS6] for details). Additionally, the pulses were treated with a wideband, uniform rate, smooth truncation (WURST) amplitude modulation to reduce Fourier transform (FT) artifacts caused by the pulse edges [Bibr bib1.bibx39]. Compensation of the nonlinearity of the SSPA yielded the final pulse shape. These pulse shape modifications are also implemented in the pulse function of MATLAB's EasySpin toolbox [Bibr bib1.bibx59]. Experimental inversion profiles of representative chirp pulses demonstrated that these pulses could achieve broadband excitation and inversion (Fig. [Fig App1.Ch1.S1.F14]).

We are aware that there are multiple possibilities for further optimizing the chirp pulses [Bibr bib1.bibx21]. Most importantly, we measured the resonator profile only once and at a different temperature than used for most of the experiments shown in this work. Compensation based on this profile will lead to imperfections as the resonator profile is dependent on temperature and tuning. Furthermore, there are other more advanced compensation schemes that take into account the complete impulse response function of the spectrometer and not just its amplitude response [Bibr bib1.bibx19]. However, [Bibr bib1.bibx18] showed that the effect of the latter is usually small. As this work is constrained to narrow-lined EPR samples where possible distortions caused by the chirp pulses are limited, we refrained from any further correction steps. Nevertheless, we expect that improved corrections might become necessary for samples with larger spectral widths.

### 3.5 CHEESY experiments

Chirp echo EPR spectroscopy (CHEESY) experiments were set up following the Böhlen–Bodenhausen scheme. Namely, 1 and 0.5µs chirp pulses were used as π/2 and π pulses, respectively, to achieve simultaneous refocusing of spins with different resonance frequencies [Bibr bib1.bibx8]. An eight-step phase cycle of the chirp pulses ensured that only the desired signal was detected (see Sect. [Sec App1.Ch1.S2.SS4]). BDPA spectra were measured at a frequency offset $\nu_{\mathrm{off}}\approx -30\;\mathrm{MHz}$ to avoid zero-frequency artifacts from LO leakage and baseline imperfections. The Rabi frequency of the chirp pulses was optimized by chirp nutations. For this purpose, the intensity and shape of the Fourier-transformed chirp echo were monitored as a function of $\nu_{1}$ [Bibr bib1.bibx17]. The $\nu_{1}$ value where the integral of the FT spectrum was maximal was chosen as the optimal setting. To measure the spectral intensity across the whole 200MHz excitation bandwidth of the chirp pulses, a frozen sample of 4-hydroxy-2,2,6,6-tetramethylpiperidinyloxyl (TEMPOL) in H_2_O / glycerol was used as it yielded a broad and structured FT spectrum. First, the Rabi frequency of the π/2 pulse was optimized using a non-optimized π pulse. The experiment showed an optimum at $\nu_{1}=7.4\;\mathrm{MHz}$ which, integrated over the resonator profile, corresponded to $Q_{\mathrm{crit}}\approx 0.6$ . This value was close to the theoretical optimum of $Q_{\mathrm{crit}}=2\mathrm{ln}(2)/\pi \approx 0.44$ predicted by [Bibr bib1.bibx36] for a π/2 chirp pulse (see Fig. [Fig App1.Ch1.S1.F15]a). Second, the optimized $\nu_{1}$ was set for the π/2 pulse, and the power of the π pulse was varied in the same way. The Rabi frequency with maximal signal was $\nu_{1}\approx 5.6\;\mathrm{MHz}$ . Considering that this pulse was shorter than the π/2 pulse, its low optimal MW intensity was surprising. As discussed by [Bibr bib1.bibx36], this effect can be attributed to a combination of a dynamic $Q_{\mathrm{crit}}$ -dependent phase shift and $\nu_{1}$ inhomogeneity. This observation suggested that the inversion efficiency of the pulse was low, presumably causing significant reduction of echo signal. Despite these nonideal conditions, we could achieve good agreement between the chirp echo FT spectrum of BDPA and the corresponding echo-detected (ED) EPR experiment (Fig. [Fig App1.Ch1.S1.F15]b). This demonstrated that we can detect narrow-lined EPR spectra using FT spectroscopy without creating distortions or artifacts. Additionally, we measured the offset dependence of the FT BDPA spectrum, which showed that the FT intensity decreases with larger frequency offsets, approximately following the shape of the resonator profile (Fig. [Fig App1.Ch1.S1.F15]c, d). Similarly, the broad EPR spectrum of TEMPOL was perturbed by the FT approach. Within the excitation bandwidth of the chirp pulses, the approximate convolution of the EPR spectrum and the resonator profile was obtained, with some additional distortions caused by pulse imperfections (see Fig. [Fig App1.Ch1.S1.F16]).

CHEESY experiments to determine pulse inversion profiles of rectangular pulses consisted of the analyzed pulse, followed by the optimized chirp echo. To generate a hole profile narrower than the EPR spectrum of BDPA, a low-power MW pulse ( $\nu_{1}\approx 0.7\;\mathrm{MHz}$ ) was used. The CHEESY spectra were obtained by subtracting the background echo without an initial pulse from the detected signal. The spectra were normalized to the background, and unity was added to afford the $z^{\prime }$ magnetization profiles, as demonstrated by [Bibr bib1.bibx66]. In contrast to the detection of pulse profiles with ELDOR [Bibr bib1.bibx31], this procedure yielded the complete hole shape in a single experiment without any parameter sweep. Similar experiments for the analysis of inversion profiles via FT were reported by [Bibr bib1.bibx61] yet without the use of chirped pulses.

### 3.6 R-CHEESY

We performed refocused (R)-CHEESY experiments with a modified pulse sequence consisting of two chirped π pulses of equal length, which refocused coherence generated by the preceding analyzed pulse. In analogy to the Böhlen–Bodenhausen scheme for the standard chirp echo, the second refocusing chirp pulse was required to refocus spins with different offsets at the same time [Bibr bib1.bibx8]. Both pulses of this sequence were set with an optimized MW power, as described in the previous section. To filter all undesired echoes, a 32-step phase cycle was used [Bibr bib1.bibx7] (see Sect. [Sec App1.Ch1.S2.SS5]). Without a background signal for reference, all spectra of one data set were normalized to the global maximum. As the FT provided a complex signal, the coherence's $x^{\prime }$ and $y^{\prime }$ components could be separated into the real and imaginary signal parts. However, in contrast to the $z^{\prime }$ profiles where phasing could be done with respect to the Gaussian line of the unperturbed BDPA spectrum, the $x^{\prime }y^{\prime }$ profiles only contained the coherence generated by the analyzed pulse. This prevented an absolute phase determination, as there was no reference phase for the data processing. Therefore, the phase was adjusted for the individual FT spectra to maximize the absorptive (i.e., axially symmetric to zero) signal in the real channel. In this way, we achieved agreement with simulations (vide infra).

### 3.7 Fourier transformation of chirp echoes

Chirp echoes obtained from CHEESY spectroscopy were corrected for offset with a zeroth-order baseline correction. A symmetric FT window around the echo signal was chosen, and the experimental signal was apodized with a cosine window function. The result was filled with zeros, with the number of zeros equal to the number of data points, and shifted using the fftshift command in MATLAB. This time-domain signal was Fourier transformed using the fast Fourier transformation algorithm implemented in MATLAB. Subsequently, a constant phase correction with $\phi_{0}$ was applied to afford the phased, complex FT spectrum. If not denoted otherwise, $\phi_{0}$ was selected to maximize the absorptive contribution in the signal's real part.

For the CHEESY $z^{\prime }$ profiles, this process could be automated using a home-written MATLAB routine. In the case of R-CHEESY, the FT window had to be selected manually for each data set, because the automatized selection of the time-domain signal maximum was unstable. If not adjusted manually, this led to linear phase drifts in the resulting FT spectra.

### 3.8 Spin density matrix simulations

The home-written simulations reported in this work were performed based on the density operator formalism in Liouville space. The initial density operator ${\hat{\rho }}^{\prime }(0)$ for a S=1/2 electron spin system was defined according to Sect. [Sec Ch1.S2.SS2]. The Hilbert space Hamiltonian for each piecewise constant element of the simulated sequence was set up in the selected frame of reference. If necessary, ${\hat{\rho }}^{\prime }(0)$ was transferred to the same reference frame. Both the initial density matrix and the Hamiltonians were then transformed into their respective Liouville space representations, and the latter was used to calculate the corresponding propagators (Eqs. [Disp-formula Ch1.E11]–[Disp-formula Ch1.E13]). The density matrix was propagated by consecutive application of the propagation superoperators (Eq. [Disp-formula Ch1.E13]), and the final output was obtained as the expectation value of the Cartesian operator of interest. Simulation parameters were set in agreement with their experimental counterparts. 

SL Rabi nutations (Sect. [Sec Ch1.S4.SS1.SSS1]) and SL echoes (Sect. [Sec Ch1.S4.SS1.SSS2]) were calculated in the rotating frame using ${\hat{\rho }}^{\prime }(0)={\hat{S}}_{y^{\prime }}$ or ${\hat{S}}_{z^{\prime }}$ for SL and without-preparation (WOP) SL experiments, respectively, as well as assuming $\Omega_{S}=0$ . While the delays between the PM pulses were simulated in a single step using ${\hat{H}}^{\prime }$ as defined in Eq. (3), the PM pulses were incremented with a step size Δt=1ns as ${\hat{H}}_{\mathrm{PM}}^{\prime }$ is time dependent. In this way, a constant Hamiltonian could be assumed within these increments, as demonstrated by [Bibr bib1.bibx42]. Relaxation was neglected during these calculations. For each value of $t_{\mathrm{PM}}$ or $\tau_{2}$ , the result was obtained as the expectation value $\langle {\hat{S}}_{y^{\prime }}\rangle$ (see Fig. [Fig Ch1.F2]c). In the case of the SL echo experiments, MW inhomogeneity was included in the SL echo simulations by repeating the calculation for different $\nu_{1}$ values. The sum of these individual traces weighted with an experimental $\nu_{1}$ profile yielded the depicted simulation results (details in Sect. [Sec App1.Ch1.S1.SS5]). To calculate the effect of PC, the simulations were repeated with different phases $\phi_{\mathrm{PM}2}$ , and the results were combined in the same way as in the experiment.

The CHEESY profiles were simulated starting from ${\hat{\rho }}^{\prime }(0)={\hat{S}}_{z^{\prime }}$ . A linear array of spin offsets $\Omega_{S}/2\pi$ was generated corresponding to the experimental $\nu -\nu_{\mathrm{off}}$ axis. For each value of $\Omega_{S}$ , the initial density matrix ${\hat{\rho }}^{\prime }(0)$ was transformed into the tilted eigenframe of the MW Hamiltonian ${\hat{H}}^{\prime }$ (Eqs. 3, 7, and 8). The final state after a pulse with length $t_{p}$ was calculated using a single propagator ${\hat{\hat{U}}}^{\prime \prime }$ calculated from ${\hat{H}}^{\prime \prime }$ (Eq. [Disp-formula Ch1.E9]) and including $T_{2\rho }$ relaxation (see Eqs. [Disp-formula Ch1.E14] and [Disp-formula Ch1.E15]). Again, MW inhomogeneity was included as the weighted sum of the results for different $\nu_{1}$ values using the experimental $\nu_{1}$ profile shown in Sect. [Sec App1.Ch1.S1.SS5]. After back-transformation of ${\hat{\rho }}^{\prime }(0)$ into the rotating frame, the final result was obtained as the expectation values $\langle {\hat{S}}_{z^{\prime }}\rangle$ for the CHEESY $z^{\prime }$ profiles and as $\langle {\hat{S}}_{x^{\prime }}\rangle$ and $\langle {\hat{S}}_{y^{\prime }}\rangle$ for the $x^{\prime }y^{\prime }$ profiles (see Fig. [Fig Ch1.F5]e).

### 3.9 Spinach simulations

Simulations using the Spinach library [Bibr bib1.bibx29] were done based on an adapted version of the holeburn.m function. The CHEESY spectrum was calculated for an anisotropically broadened powder EPR line (see Sect. [Sec App1.Ch1.S1.SS14]) in three steps. First, the initial equilibrium spin system was propagated under a soft MW SL pulse including transversal relaxation, in the tilted frame. Second, an ideal, non-selective π/2 rotation was applied that transformed all residual $z^{\prime }$ components into coherences. Third, simulation of the powder-averaged free induction decay (FID) and FT yielded the CHEESY spectrum. In order to filter the FID of the SL pulse, a two-step PC of the SL was applied in the simulations (i.e., $\phi_{\mathrm{SL}}=[0{}^{\circ},180{}^{\circ}]$ and $\phi_{\det}=[+,+]$ ). Due to the increased computational cost of the calculations, $\omega_{1}$ inhomogeneity was not included. The results were compared to calculations using the unmodified holeburn.m function and rotating frame relaxation implemented as t1_t2.m (see Sects. [Sec Ch1.S4.SS2.SSS1] and [Sec App1.Ch1.S1.SS14]).

## 4 Results

### 4.1 Spin-locked EPR experiments

We performed SL EPR experiments to probe the spin dynamics during MW irradiation.

#### 4.1.1 SL Rabi nutations

Figure [Fig Ch1.F2]a shows the pulse sequence for SL Rabi nutation experiments, which can be used to determine the optimal PM pulse lengths for further SL EPR experiments. The sequence consists of an initial rectangular π/2 pulse ( $x^{\prime }$ ) that prepares electron coherence, which is subsequently locked by the SL pulse along $y^{\prime }$ . The preparation and SL pulses were performed with $\nu_{1}=15\;\mathrm{MHz}$ , which was considerably less than the 100MHz used by [Bibr bib1.bibx67] at 34 GHz and not sufficient to lock the entire BDPA spectrum with a width of ≈24MHz in the transversal plane. The effects of this will be discussed later in this section.

**Figure 2 Ch1.F2:**
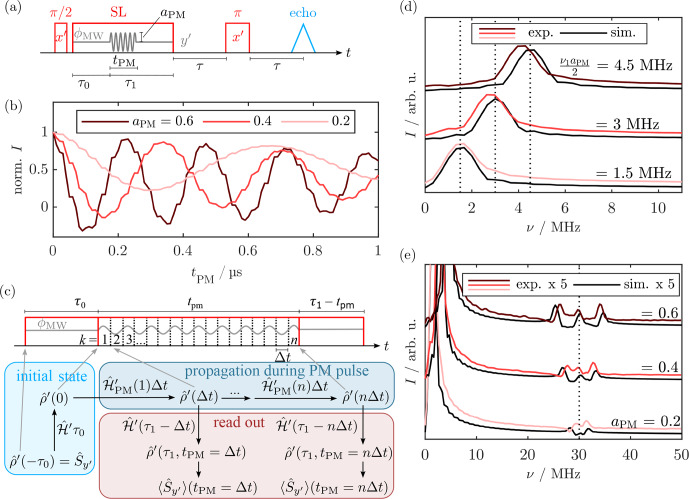
**(a)** Pulse sequence for the SL Rabi nutation. The echo was integrated as a function of the PM pulse length $t_{\mathrm{PM}}$ . **(b)** Experimental SL Rabi nutation traces for $\nu_{1}=15\;$ MHz with different modulation amplitudes $a_{\mathrm{PM}}$ . **(c)** Simulation approach for the SL Rabi nutation experiments by stepwise evolution of the rotating frame density operator during a phase-modulated pulse. **(d, e)** FT spectra of the experimental nutation traces (red) in the low-frequency region **(d)** and enlarged in the high-frequency region **(e)**. Simulated nutations are shown in black, accurately reproducing the experimental peak positions. In **(d)**, dotted lines mark the positions where a signal is expected for the respective SL Rabi frequency $\nu_{1}\cdot a_{\mathrm{PM}}/2$ . The dotted line in **(e)** marks $\nu =2\nu_{1}$ . For experimental parameters, see Sect. [Sec App1.Ch1.S2.SS1].

During SL and after an initial SL delay $\tau_{0}$ , a PM pulse was applied by periodic modulation of the SL phase for a variable length $t_{\mathrm{PM}}$ and with a modulation amplitude $a_{\mathrm{PM}}$ . The overall SL length $\tau_{0}+\tau_{1}$ was constant. The effect of the PM pulse was monitored by a bare-state (rotating frame) echo produced by a π pulse after a delay τ that refocused coherence in the rotating frame at 2τ after the SL pulse. Figure [Fig Ch1.F2]b shows the SL Rabi nutation traces for three different modulation amplitudes $a_{\mathrm{PM}}$ . The SL Rabi traces contained a dominant slow oscillation with a superimposed weaker and faster modulation. FT of the time traces (Fig. [Fig Ch1.F2]d) showed that the dominant, slower oscillation corresponded to a SL Rabi frequency of $\nu_{1}a_{\mathrm{PM}}/2$ . This agrees with the theoretical predictions from the nutating frame transformation (Eq. [Disp-formula Ch1.E6]). The faster oscillation frequency appeared at $2\nu_{1}$ plus two sidebands (Fig. [Fig Ch1.F2]e). We simulated the experiments using density matrix calculations (see Fig. [Fig Ch1.F2]c and Sect. [Sec Ch1.S3.SS8]), neglecting a bare-state offset by setting $\Omega_{S}=0$ . Figure [Fig Ch1.F2]d and e show almost quantitative agreement between the Fourier transformed experimental and simulated time traces. Interestingly, the fast oscillation also directly resulted from the simulation. We attribute this effect to a deviation from the RWA, leading to a minor yet visible modulation with the counter-rotating wave that precesses with $2\nu_{1}$ . The simulations also qualitatively reproduced the sidebands of $2\nu_{1}$ , which might be caused by frequency mixing. Comparison with the results by [Bibr bib1.bibx42] shows that they reported similar fast frequency oscillations in their simulation for tilted-frame excitation by modulation of the frequency offset instead of phase modulations.

This good agreement without simulating any bare-state offset was unexpected, as the strong driving condition $\omega_{1}\gg \Omega_{S}$ is not met in our experiments. Simulations including a bare-state offset showed that severe distortions occurred for a single, sharp offset $\Delta \Omega >\omega_{1}$ (see Fig. [Fig App1.Ch1.S1.F18]a). However, simulating a Gaussian distribution of $\Omega_{S}$ values that was centered at 0MHz yielded a nearly unperturbed FT spectrum (Fig. [Fig App1.Ch1.S1.F18]b). This suggests that offset effects are averaged during the experiment, so the final spectrum agrees with the predictions from the ideal $\omega_{1}\gg \Omega_{S}$ case.

**Figure 3 Ch1.F3:**
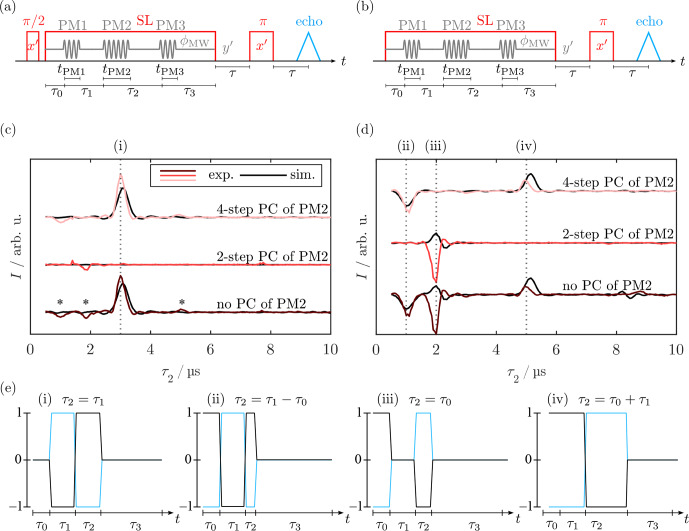
Pulse sequence for standard **(a)** and WOP **(b)** SL echoes. Real components of the quadrature detection signal of the SL echo **(c)** and WOP SL echo **(d)** spectra measured with different phase cycles. The detection phase was set to maximize $y^{\prime }$ magnetization in the real channel. Experimental traces are in different shades of red, and corresponding simulations are in black. Signals (i)–(iv) were assigned in **(e)** and belong to evolution pathways that are refocused at the beginning of PM3. In **(c)**, the asterisks denote the residual signals (ii), (iii), and (iv). The imaginary part of the spectra with signals refocused at the end of $\tau_{3}$ can be found in Fig. [Fig App1.Ch1.S1.F19]. For experimental parameters, see Sect. [Sec App1.Ch1.S2.SS2].

#### 4.1.2 SL echoes

In the next step, we examined the feasibility of observing spin echoes during SL. Using a PM π/2 pulse followed by a delay and a PM π pulse, the analog of a Hahn echo can be created during SL [Bibr bib1.bibx67]. The refocused SL coherence is read out by a third PM π/2 pulse that realigns the magnetization with the SL field, which can be refocused with a bare-state echo (see Fig. [Fig Ch1.F3]a). We monitored the refocusing of SL coherence by variation of the delay $\tau_{2}$ between the refocusing (PM2) and readout pulse (PM3). The SL pulse length was kept constant by simultaneous variation of $\tau_{3}$ . For $\tau_{2}=\tau_{1}=3\;µs$ , refocusing was observed as a strong echo (Fig. [Fig Ch1.F3]c), which indicates that PM experiments are feasible under W-band experimental conditions, i.e., weaker MW pulses and considerable offset contributions. In addition to this, weak signals (Fig. [Fig Ch1.F3]c, asterisks) whose position depended on the length of the delay $\tau_{0}$ were observed. According to [Bibr bib1.bibx67], these are caused by incomplete preparation of the coherent state $S_{y^{\prime }}$ , leaving spins with residual $S_{z^{\prime }}$ components unaligned with the spin lock field.

In the same way as the π/2 preparation pulse, the PM pulse rotation angles can deviate from the desired π/2 and π values, so several signals can be produced by different effective rotation angles of the individual PM pulses. We used two- and four-step phase cycling (PC) of the phase-modulated inversion pulse PM2 to eliminate the small crossing echoes and two-step phase cycling of the phase-modulated readout pulse PM3 to filter non-locked components. A two-step PC of PM2 ( $\phi_{\mathrm{PM}2}=[0{}^{\circ},180{}^{\circ}]$ ) with opposite detection phase ( $\phi_{\det}=[+,-]$ ) selects coherence transfer pathways in which PM2 changes the coherence order by one, i.e., only when it acts as a π/2 pulse. In contrast, a four-step PC of PM2 ( $\phi_{\mathrm{PM}2}=[0{}^{\circ},90{}^{\circ},180{}^{\circ},270{}^{\circ}]$ ) with alternating detection phase ( $\phi_{\det}=[+,-,+,-]$ ) selects a transfer pathway, where PM2 causes a change in coherence order by two, i.e., where it acts as a π pulse. As expected, the two-step PC of PM2 eliminated the main echo signal, whereas it was retained by the four-step PC (see Fig. [Fig Ch1.F3]c). Additionally, the small crossing echoes were affected by the different phase cycles, demonstrating that they arise from different coherence transfer pathways.

As incomplete preparation is supposed to be significant under W-band conditions, we analyzed the SL coherence pathways by omitting the π/2 preparation step. This type of experiment without preparation (WOP) was already implemented for CP-ENDOR by [Bibr bib1.bibx4], but the spin dynamics were not reported. Figure [Fig Ch1.F3]b depicts the modified pulse sequence, and panels (c) and (d) show the comparisons of the SL echo with and without preparation obtained by variation of $\tau_{2}$ while keeping $\tau_{0}=2\;µs$ and $\tau_{1}=3\;µs$ fixed. Both experiments were performed using the same experimental parameters.

The WOP SL echo experiment produced signals at 1µs (ii), 2µs (iii), and 5µs (iv) (Fig. [Fig Ch1.F3]d), which differs significantly from those observed in (c). Notably, the primary echo (i) is missing, while the observed echo positions coincide with the artifacts observed in (c). To rationalize this observation, we analyzed the refocusing times $\tau_{2}$ and the effect of PC. The strongest WOP SL echo (iii) is observed at $\tau_{2}=\tau_{0}$ , suggesting that it is created by a coherence that starts to evolve during $\tau_{0}$ and refocuses as a stimulated echo. This is corroborated by the PC, as the signal is retained by a two-step PC of PM2 and vanishes upon four-step PC. With the same rationale, signals (ii) and (iv) can be assigned to a refocused echo and Hahn echo, respectively. Coherence diagrams visualizing the idealized evolution pathways (i.e., $\Omega_{S}=0$ ) responsible for each signal are shown in Fig. [Fig Ch1.F3]e, along with their respective refocusing condition. These results are consistent with the spins having a Boltzmann distribution in the rotating frame corresponding to $z^{\prime }$ magnetization prior to SL. Upon start of the SL pulse, i.e., under MW irradiation, this becomes a coherence in the tilted frame, which immediately evolves with $\nu_{1}$ .

The peak positions (i)–(iv) as well as their line width and the effect of PC could be reproduced by density matrix simulations following the procedure described in Sect. [Sec Ch1.S3.SS8] and Fig. [Fig Ch1.F2]c. The phase and intensity of the WOP signals agreed only partially. Repetition of the experiment under nominally the same conditions showed that the phase of the individual signals was not reproducible. Detection of the out-of-phase component by setting $\phi_{\mathrm{PM}3}=\pi /2$ underlined that there is a phase instability for the WOP echoes (see Sect. [Sec App1.Ch1.S1.SS9]). We suppose that this is the case because the turning angles of the PM pulses were not optimized for these specific coherence pathways.

Altogether, our results are consistent with the assumption that equilibrium $z^{\prime }$ magnetization becomes a coherence during MW irradiation. Hence, these results show that, under our experimental conditions, conventional spin locking with a preparation step and long MW pulses acting on spins initially in thermal equilibrium are closely related. By application of PM pulses, the spin states can be transferred from SL coherence to SL populations and vice versa.

#### 4.1.3 Relaxation during spin lock

To measure relaxation times during SL, the delays $\tau_{1}$ and $\tau_{2}$ of the SL echo sequence (see Fig. [Fig Ch1.F3]a) were both increased so that refocusing was achieved throughout the experiment, while the evolution time in the transverse plane was increased. Simultaneously, $\tau_{3}$ was decreased to ensure a constant SL length. The comparison of the decays obtained with $\nu_{1}=15$ MHz and the bare-state Hahn echo decay can be found in Fig. [Fig Ch1.F4]a. The experimental traces were fitted using both a mono-exponential and a stretched-exponential function. We calculated the residuals, and statistical errors were determined by bootstrapping (for details, see Sect. [Sec App1.Ch1.S1.SS10]). With this, we could compare the two decay models and evaluate the systematic error introduced when using the simplified mono-exponential model. The decay of coherence was approximately 2–3 times slower in the SL state than in the rotating frame with mono-exponential decay times of 3.6±0.2 and 1.46±0.02µs , respectively (red and blue curves in Fig. [Fig Ch1.F4]a). Fitting with a stretched-exponential decay function led to $T_{2\rho }=5.0\pm 0.3$ and $T_{m}=2.19\pm 0.03\;µs$ . Analysis of the residuals (bottom part in Fig. [Fig Ch1.F4]a) showed that there is a systematic deviation in the mono-exponential fit (red curve). Namely, the intensity is overestimated for $t_{p}<10\;µs$ and underestimated for $t_{p}>10\;µs$ . This systematic error was reduced when using the stretched-exponential fit (dark red curve), which is consistent with the literature concerned with bare-state echo decays, where stretched-exponential functions are often used. [Bibr bib1.bibx46].

**Figure 4 Ch1.F4:**
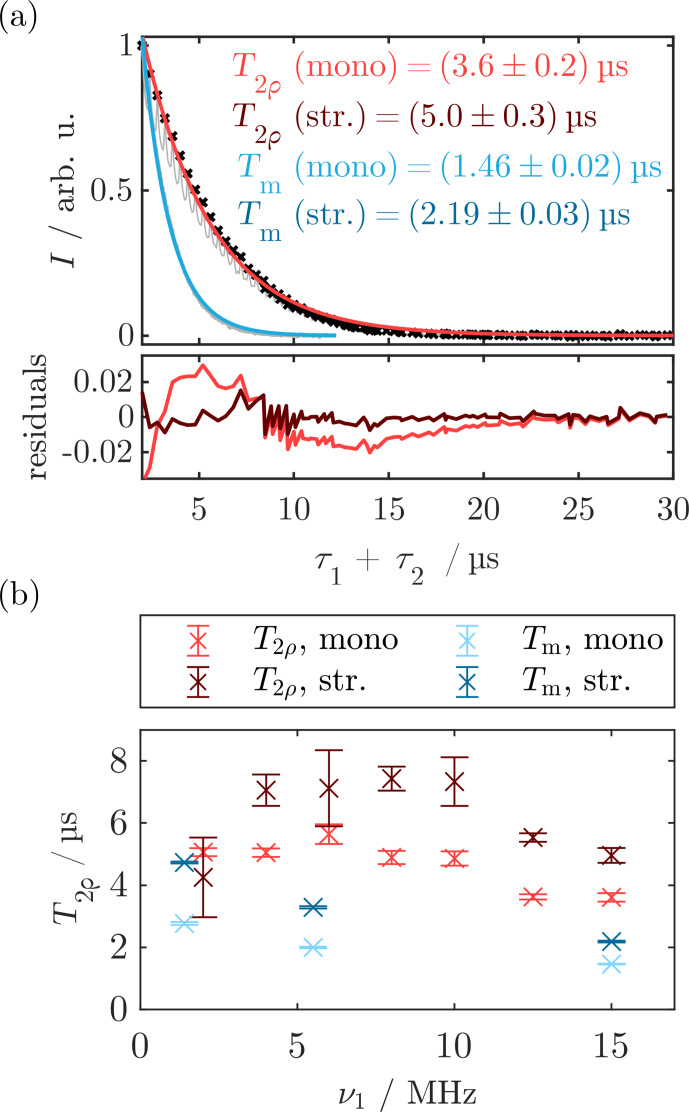
SL relaxation measurements obtained with the standard SL echo sequence as depicted in Fig. [Fig Ch1.F3] by variation of $\tau_{1}+\tau_{2}$ . **(a)** SL relaxation trace measured for $\nu_{1}=\nu_{\mathrm{PM}}=15\;\mathrm{MHz}$ (red) compared to the $T_{m}$ relaxation trace in the bare state (blue). Experimental time traces are in gray, and the envelope function used for fitting the SL state decay is depicted as black dots. Mono-exponential fits for $T_{2\rho }$ and $T_{m}$ are in red and blue, respectively. Residual of the mono-exponential fit for $T_{2\rho }$ in the bottom part (red) compared to the residuals from the stretched-exponential fitting (dark red). **(b)** $T_{2\rho }$ data measured for different SL powers and comparison with $T_{m}$ values obtained from Hahn echo decays with different $\nu_{1}$ values and pulse lengths. The corresponding traces and fits can be found in Fig. [Fig App1.Ch1.S1.F21]. Error bars correspond to the statistical error, while the systematic error might be larger. For experimental parameters, see Sect. [Sec App1.Ch1.S2.SS3].

Investigation of the SL power dependence of $T_{2\rho }$ (Figs. [Fig Ch1.F4]b and [Fig App1.Ch1.S1.F21]) showed no clear trend, and an overall increase in the transverse relaxation time by a factor of 2 to 4 compared to $T_{m}=1.4\;µs$ was obtained from a mono-exponential fit (red). The stretched-exponential fitting yielded $T_{2\rho }$ values between 4 and 8µs . These results show a similar trend as the low-field results by [Bibr bib1.bibx67] that were performed at much higher MW fields $\nu_{1}\approx 100\;\mathrm{MHz}$ , reporting an increase in $T_{2\rho }$ relative to $T_{m}$ by a factor of 4.5 for stretched-exponential fitting. These observations show potential for designing new pulse sequences that mitigate short decoherence times. The stretching exponents ξ for the stretched-exponential fits were between 0.9 and 1.7 (see Sect. [Sec App1.Ch1.S1.SS10]). Importantly, there is a strong correlation between the $T_{2\rho }$ value and ξ (Pearson correlation within the bootstrap samples above 0.9 for all traces). Therefore, the choice of the stretching coefficient affected the obtained decay time.

Analysis of Hahn echo decays measured with lower $\nu_{1}$ (i.e., longer and more selective pulses) revealed increased $T_{m}$ times (blue in Fig. [Fig Ch1.F4]b), indicating a contribution of instantaneous diffusion to the bare-state decoherence. Additionally, we measured the echo decay with the three- and four-pulse Carr–Purcell (CP) sequences [Bibr bib1.bibx10]. Both CP3 and CP4 led to an increase in decoherence time by around 50% relative to the two-pulse measurement with the same $\nu_{1}$ (see Sect. [Sec App1.Ch1.S1.SS11]).

Notably, the SL echo decays were perturbed by an oscillation for small $\tau_{1}+\tau_{2}$ (gray curve), which, to our knowledge, has not been reported in the literature. We attribute this to the edge effects of the PM pulses. A detailed analysis and quantitative explanation of these artifacts can be found in Sect. [Sec App1.Ch1.S1.SS12] but is of little consequence for the following results. We circumvented this effect by fitting the envelope function of the time trace (black dots in Fig. [Fig Ch1.F4]a). This method is also used to analyze Hahn echo decay traces perturbed by electron spin echo envelope modulations (ESEEM) [Bibr bib1.bibx57]. However, the periodic oscillations in the residuals for $\tau_{1}+\tau_{2}<10\;µs$ are indicative of small artifacts caused by this envelope fitting. Overall, these experiments provided us with an estimate for the decoherence time during SL over a broad range of MW powers. In the following sections, this will be used to model the spin dynamics during MW irradiation by calculating trajectories of our S=1/2 model system. For this, a transverse relaxation time of $T_{2\rho }\approx 4\;µs$ will be assumed as an approximation of the mono-exponential $T_{2\rho }$ time. Due to the strong correlation of $T_{2\rho }$ and ξ as well as the limitation of our simulation method to mono-exponential decays, we refrain from using the stretched-exponential results for the following calculations.

### 4.2 CHEESY detection of pulse excitation profiles

CHEESY experiments were performed to analyze the spin state immediately after long, rectangular MW pulses.

**Figure 5 Ch1.F5:**
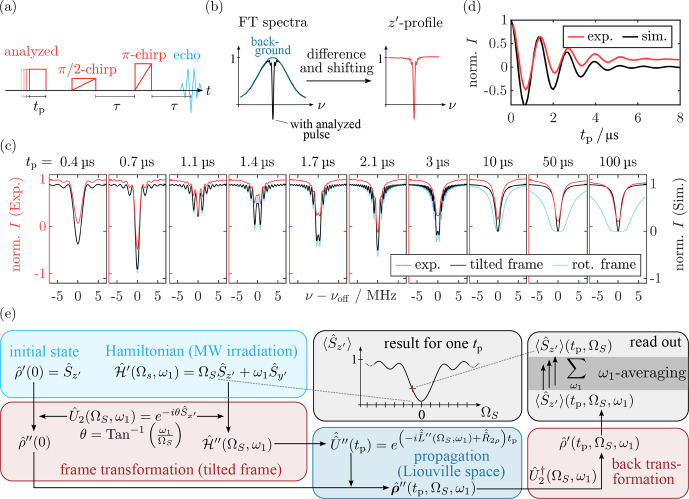
**(a, b)** CHEESY pulse sequence and schematic data processing. Inversion ( $z^{\prime }$ ) profiles were obtained by subtracting a chirp echo background spectrum without the first pulse from the normalized hole profiles and vertically shifting the results by one [Bibr bib1.bibx66]. **(c)** Experimental (red) and simulated (black) inversion profiles for a low-power MW pulse ( $\nu_{1}=0.77\;\mathrm{MHz}$ ). For better visibility, the latter are vertically shifted by -0.1 . Simulations were performed using the simulation routine depicted in **(e)** with a variation of $t_{p}$ and $\Omega_{S}$ (see Sect. [Sec Ch1.S3.SS8]). Results from an analogous simulation omitting the tilted-frame transformation are shown in light blue. **(d)** Rabi nutation obtained from CHEESY inversion profiles at $\nu -\nu_{\mathrm{off}}=0$ (red) and from the tilted-frame simulation (black). For experimental parameters, see Sect. [Sec App1.Ch1.S2.SS4].

#### 4.2.1 $z^{\prime }$ profiles

We probed the residual $z^{\prime }$ magnetization after a soft MW pulse of varied length $t_{p}$ with a chirp echo (see Fig. [Fig Ch1.F5]a). For each $t_{p}$ , FT of the chirp echo transient and subtraction of a background chirp echo without additional pulse yielded the inversion profile as a hole burned in the initial $z^{\prime }$ magnetization (Fig. [Fig Ch1.F5]b). Figure [Fig Ch1.F5]c shows the obtained hole shape as a function of $t_{p}$ (red).

For short irradiation times, the profiles were sinc-shaped, as expected for a rectangular pulse. A distinct Rabi oscillation was detected, i.e., a periodic variation of the hole depth at the center frequency $\nu -\nu_{\mathrm{off}}=0$ as a function of $t_{p}$ (Fig. [Fig Ch1.F5]d, red). FT of the Rabi oscillation trace yielded a nutation frequency $\nu_{1}=0.77\;\mathrm{MHz}$ . The magnitude of the oscillation decreased with increasing pulse length. Comparison with the experimental $T_{2\rho }$ relaxation traces and simulations (see Fig. [Fig App1.Ch1.S1.F25]) showed that this decay was faster than the SL coherence decay. We attribute this fast decay to MW inhomogeneity, resulting in a broadened distribution of nutation frequencies. Simulations including both the experimentally determined $T_{2\rho }$ relaxation rate and MW inhomogeneity could quantitatively reproduce the Rabi trace (black in Fig. [Fig Ch1.F5]d; see Sect. [Sec App1.Ch1.S1.SS13]). For long MW pulses with $t_{p}≳10\;µs$ , a steady state was reached where the excitation profile no longer depended on $t_{p}$ and resembled a Lorentzian-shaped saturation profile with FWHM≈1.6MHz . This saturation hole shape agrees with observations of the central hole in standard EDNMR experiments with rectangular high-turning angle pulses and a full width at half maximum of $2\nu_{1}$ [Bibr bib1.bibx47]. The simulations also reproduced the transition from a $t_{p}$ -dependent, sinc-shaped hole to a steady-state Lorentzian as well as the excitation bandwidth. Moreover, the dampening of the Rabi oscillation curve was simulated quantitatively. Crucially, the simulation results changed when the transformation in the tilted frame was omitted. In this case, the simulations for long $t_{p}$ no longer matched the experiment, and an increased width of the simulated hole was observed (light blue in Fig. [Fig Ch1.F5]c). We attribute this to the fact that, if not in the eigenstate of the full MW Hamiltonian, the relaxation term in the Liouville space propagator does not act on pure coherences but on mixed coherence and population density matrix elements. This leads to incorrect dampening of population terms during decoherence.

The most apparent difference between experimental and simulated profiles in the tilted frame is the resolution in the frequency dimension, i.e., the degree to which the narrow sinc lobes for intermediate pulse lengths are resolved. While the oscillations are clearly visible in the simulations up to $t_{p}=3\;µs$ , they are hardly visible in the experimental spectra with $t_{p}>1.6\;µs$ . This is presumably caused by the intrinsic experimental FT resolution limit posed by the finite FT window during data processing.

**Figure 6 Ch1.F6:**
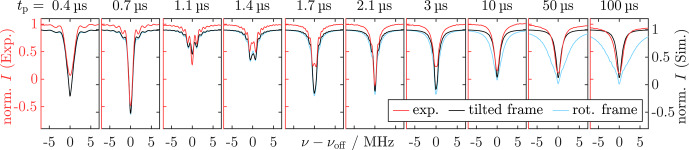
CHEESY $z^{\prime }$ profiles simulated with the Spinach library [Bibr bib1.bibx29] using propagation in the tilted frame (black) and comparison with the experimental profiles (red). For better visibility, the simulations are vertically shifted by -0.1 . Analogous calculations in the rotating frame using the holeburn.m function are in light blue. For experimental parameters, see Sect. [Sec App1.Ch1.S2.SS4].

We compared our results to simulations using the Spinach library [Bibr bib1.bibx29], where we explicitly calculated the hole burning in a powder-averaged EPR spectrum by Fourier transformation of a time-domain CHEESY signal (see Sects. [Sec Ch1.S3.SS9] and [Sec App1.Ch1.S1.SS14]). As in the home-written simulation routine, transformation in the tilted frame during propagation of the SL pulse was necessary to reproduce the experimental results (compare black and light blue lines in Fig. [Fig Ch1.F6]). Compared to Fig. [Fig Ch1.F5], the line width agreed more accurately because the FT was explicitly included in the simulation (see Sect. [Sec Ch1.S3.SS9]). However, the dampening of the Rabi oscillation obtained from the intensity at $\nu -\nu_{\mathrm{off}}=0$ was not quantitatively reproduced (compare Figs. [Fig App1.Ch1.S1.F26] and [Fig Ch1.F5]d), because increased computational time prevented incorporation of MW inhomogeneity.

Comparison of tilted-frame simulations with different $T_{2\rho }$ values showed that using a shorter relaxation time $T_{2\rho }=1.4\;µs$ could not reproduce the data as the decay of the side lobes was too fast. Using a longer relaxation time $T_{2\rho }=10\;µs$ did not affect the visual shape of the pulse profiles. In contrast, completely omitting $T_{2\rho }$ could not reproduce the experiment as no steady state was reached, even for $t_{p}=100\;µs$ (see Fig. [Fig App1.Ch1.S1.F27]). So far, our simulations neglected hyperfine coupling. This is a strong simplification, as nuclear coupling, in particular its pseudo-secular component, is known to introduce additional evolution pathways during irradiation [Bibr bib1.bibx33]. Using the Spinach implementation, nuclear coupling could be introduced straightforwardly. Simulation with up to four coupled protons with A<10MHz led to no change in the CHEESY profiles when using an appropriately expanded relaxation superoperator in the tilted frame (see Fig. [Fig App1.Ch1.S1.F28]c).

Overall, the Spinach simulations validated the home-written density matrix simulations by demonstrating that, under our conditions, including nuclear couplings has no visible effect on the $z^{\prime }$ profiles. At the same time, they highlighted that omitting MW inhomogeneity leads to an underestimation of the Rabi nutation decay. Including the FT step in the simulations improved the agreement between experimental and simulated $z^{\prime }$ profiles and allowed for a qualitative assessment of the effect of $T_{2\rho }$ on reaching dampening of the sinc-shaped features of the profiles. However, the Spinach simulation requires longer computation time, preventing fast calculation and inclusion of MW inhomogeneity. This is the advantage of the home-written density matrix simulation, which we used as the main computational tool in the following.

#### 4.2.2 $x^{\prime }y^{\prime }$ profiles

The standard CHEESY approach is limited to observing $z^{\prime }$ magnetization and hole-burning profiles. To observe the coherence generated by a MW pulse, we used a modified chirp sequence where the chirp echo was replaced with two chirped π pulses that form, together with the analyzed pulse, a refocused echo sequence (R-CHEESY; see Fig. [Fig Ch1.F7]a). This experiment is closely related to the ABSTRUSE sequence developed by [Bibr bib1.bibx9]. FT of the hereby generated chirp echo yielded a complex signal. Its phase was adjusted so that the absorptive signal was maximized in the real component.

The results of the R-CHEESY experiment are shown in Fig. [Fig Ch1.F7]c (red). Oscillating components were observed for short pulse lengths $t_{p}$ that changed to a steady state for longer irradiation in the same manner and timescale as observed in the CHEESY $z^{\prime }$ profiles. Monitoring the intensity of the real component at $\nu -\nu_{\mathrm{off}}=0$ showed a continuous Rabi oscillation (Fig. [Fig Ch1.F7]d). FT of this trace yielded the Rabi frequency $\nu_{1}=0.63\;\mathrm{MHz}$ (these experiments were performed with slightly different tuning of the microwave cavity and different power settings compared to the $z^{\prime }$ profiles in the previous section). After long MW irradiation ( $t_{p}\gg T_{2\rho }$ ), the steady state exhibited negligible signal in the real part, yet a clear dispersive signal was detected in the imaginary component. This corresponded to no detectable magnetization perpendicular to the MW axis but to a distinct magnetization component parallel to $\nu_{\mathrm{eff}}$ . To rationalize this, we simulated the $x^{\prime }y^{\prime }$ profiles using the procedure described in Fig. [Fig Ch1.F5]e and Sect. [Sec Ch1.S3.SS8]. Again, there was a good agreement between experimental and simulated data for both the initial Rabi oscillation period and the steady state (Fig. [Fig Ch1.F7]c, d, black). As in the experiment, no contribution remained in the real part of the steady-state R-CHEESY signal, while a dispersive Lorentzian line was retained in the imaginary part. This can be explained by the $T_{2\rho }$ relaxation that leads to a decay of coherence in the tilted frame, i.e., of magnetization in the $x^{\prime \prime }y^{\prime \prime }\approx x^{\prime }z^{\prime }$ plane. Therefore, only the component parallel to $\nu_{\mathrm{eff}}$ which decays with the longer $T_{1\rho }$ is visible in the steady state.

**Figure 7 Ch1.F7:**
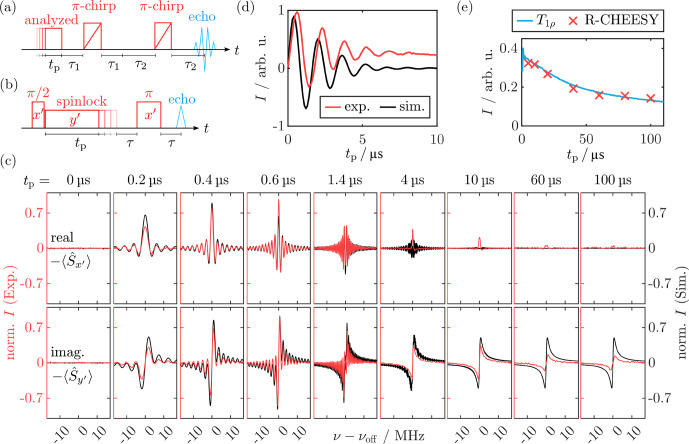
**(a)** R-CHEESY pulse sequence. **(b)** Pulse sequence for measuring longitudinal SL relaxation $T_{1\rho }$ . **(c)** Experimental (red) and simulated (black) R-CHEESY spectra for different $t_{p}$ values with their real and imaginary components. **(d)** Experimental (red) and simulated (black) Rabi nutation obtained from the R-CHEESY excitation profiles for varied pulse lengths $t_{p}$ as the real intensity at $\nu -\nu_{\mathrm{off}}=0$ . FT of the experimental data yielded $\nu_{1}=0.63\;\mathrm{MHz}$ . **(e)** Intensity of the dispersive steady-state signal from **(c)** and comparison with the $T_{1\rho }$ trace measured with $\nu_{1}\approx 0.8\;\mathrm{MHz}$ . For experimental parameters, see Sect. [Sec App1.Ch1.S2.SS5].

This is further validated by the decay of the experimental steady-state signal along $y^{\prime }$ for $t_{p}≳10\;µs$ . Overlay of the maximum $y^{\prime }$ signal intensity as a function of $t_{p}$ with a $T_{1\rho }$ decay measured at a similar spin lock power showed good agreement (see Fig. [Fig Ch1.F7]b and e). Thus, this decay could be attributed to $T_{1\rho }$ relaxation. As no longitudinal relaxation was included in our theoretical framework, this effect was not reproduced in the simulations. Simulating this effect is complicated by the fact that standard longitudinal relaxation ultimately approaches a non-zero equilibrium distribution. How to account for this in the case of $T_{1\rho }$ is disputed in the literature and goes beyond the scope of this work (e.g., p. 243; p. 316). Development of a simulation procedure and further benchmark experiments should instead be the objective of future work.

**Figure 8 Ch1.F8:**
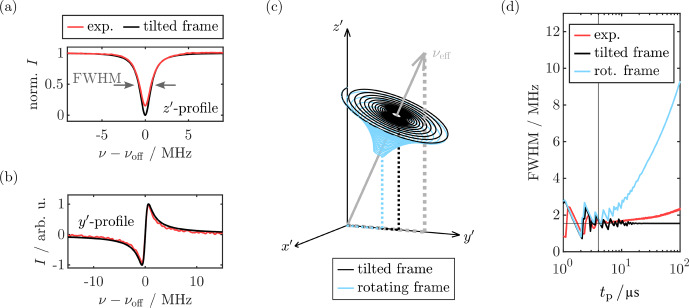
**(a, b)** Experimental steady-state $z^{\prime }$ and $y^{\prime }$ profiles ( $t_{p}=10\;µs$ ) and analytical solution from Eq. [Disp-formula Ch1.E17]. Experimental MW powers (**a**: $\nu_{1}=0.77$ MHz, **b**: $\nu_{1}=0.63$ MHz) were used for calculating the analytical solution. **(c)** Evolution of M(t) calculated analytically from the tilted-frame solution of the Bloch equations (Eq. [Disp-formula Ch1.E16], black) and comparison with the numerical solution for the standard Bloch equation in the rotating frame (Eq. [Disp-formula App1.Ch1.S1.E36], light blue). Effective MW field vector $\nu_{\mathrm{eff}}$ is shown as a gray vector, and its components $\nu_{1}=0.77$ and $\nu -\nu_{\mathrm{off}}=1.7\;\mathrm{MHz}$ are shown as dotted gray lines along $y^{\prime }$ and $z^{\prime }$ ; the $y^{\prime }$ and $z^{\prime }$ components of $M(t_{p})$ are shown as black and light blue dotted lines after $t_{p}=10\;µs$ for the tilted- and rotating-frame solutions, respectively. **(d)** FWHM of the calculated inversion profiles using both the tilted- (black) and the rotating-frame (light blue) expression as a function of $t_{p}$ . The horizontal and vertical lines mark the values $\mathrm{FWMH}=2\nu_{1}=1.54\;\mathrm{MHz}$ and $t_{p}=T_{2\rho }=4\;µs$ , respectively. Oscillations for small $t_{p}$ are caused by the oscillating side lobes of the initial sinc-shaped profiles. For additional parameters, see Sect. [Sec App1.Ch1.S2.SS6].

#### 4.2.3 Comparison with Bloch equations

As we assume an isolated S=1/2 electron spin, in principle, one can represent the spin system by a magnetization vector in three-dimensional Cartesian space and calculate its evolution from the Bloch equations [Bibr bib1.bibx6]. Being defined in the rotating frame, this is analogous to the density matrix calculations without transformation into the tilted frame. Thus, it leads to errors when implementing relaxation in the case of long MW pulses (see below). [Bibr bib1.bibx51] proposed a modification of the Bloch equations in the limit of strong irradiation in which the $x^{\prime }y^{\prime }$ relaxation was divided into a fast process perpendicular to the irradiation field and a slower one for the parallel component. Indeed, this modification resulted in line narrowing compared to the steady-state solution of the standard Bloch equations under strong driving conditions [Bibr bib1.bibx54]. Although this model is similar to our tilted-frame approach, it is different in that there are now two axes with slow relaxation and that it does not directly depend on the spin offset. In contrast to this, we analyzed the Bloch propagation in the tilted frame, in analogy to our density matrix calculations. Solution of the Bloch equations transformed in the tilted frame yielded the following rotating-frame magnetization vector (for the derivation, see Sect. [Sec App1.Ch1.S1.SS15]):

16 $$M^{\prime }(t)=\left(\begin{array}{l} \mathrm{sin}(\omega_{\mathrm{eff}}t)\mathrm{sin}(\theta )M_{0}\cdot e^{-t/T_{2\rho }}\\ \left[-\mathrm{cos}(\omega_{\mathrm{eff}}t)\mathrm{sin}(\theta )\mathrm{cos}(\theta )\cdot e^{-t/T_{2\rho }}\right.\\ \left.\;\;\;+\mathrm{sin}(\theta )\mathrm{cos}(\theta )\right]M_{0}\\ \left[\mathrm{cos}(\omega_{\mathrm{eff}}t){\mathrm{sin}}^{2}(\theta )\cdot e^{-t/T_{2\rho }}+{\mathrm{cos}}^{2}(\theta )\right]M_{0} \end{array}\right).$$

For the limiting case of infinite microwave irradiation ( t→∞ ), all terms including an exponential decay vanished, and an analytical steady-state solution was obtained:

17 $$\underset{t⟶\infty }{lim}M^{\prime }(t)=\left(\begin{array}{l} 0\\ \mathrm{sin}(\theta )\mathrm{cos}(\theta )M_{0}\\ {\mathrm{cos}}^{2}(\theta )M_{0} \end{array}\right)=\left(\begin{array}{l} 0\\ \frac{\left(\nu -\nu_{\mathrm{off}}\right)\nu_{1}}{\nu_{1}^{2}+{\left(\nu -\nu_{\mathrm{off}}\right)}^{2}}\\ 1-\frac{\nu_{1}^{2}}{\nu_{1}^{2}+{\left(\nu -\nu_{\mathrm{off}}\right)}^{2}}. \end{array}\right)$$

This steady-state expression was already proposed by [Bibr bib1.bibx1] and is in agreement with the recent literature on EDNMR [Bibr bib1.bibx47]. Comparison with the experimental steady-state $y^{\prime }$ and $z^{\prime }$ profiles showed quantitative agreement (Fig. [Fig Ch1.F8]a and b; compare to Fig. [Fig Ch1.F5]c). Additionally, in contrast to the approaches by [Bibr bib1.bibx1] and [Bibr bib1.bibx47], the complete spin trajectory during a MW pulse including relaxation could be calculated analytically using Eq. ([Disp-formula Ch1.E16]). The results for a spin offset $\Omega_{S}/2\pi =1.7\;\mathrm{MHz}$ and $\nu_{1}=0.77\;\mathrm{MHz}$ are shown as a black curve in Fig. [Fig Ch1.F8]c for a pulse length $t_{p}=10\;µs$ . Starting from $M^{\prime }=(0,0,M_{0}{)}^{T}$ , the magnetization vector precesses around $\nu_{\mathrm{eff}}$ (gray arrow) and approaches a steady state ( $y^{\prime }$ and $z^{\prime }$ components marked by dashed black line) that lies on $\nu_{\mathrm{eff}}$ . The whole evolution occurs in a plane perpendicular to $\nu_{\mathrm{eff}}$ that cuts the $z^{\prime }$ axis at $M_{0}$ . Again, we compared our results to propagation in the rotating frame by numerical solution of the Bloch equations in the rotating frame (for details, see Sect. [Sec App1.Ch1.S1.SS15]). Analysis of this trajectory (Fig. [Fig Ch1.F8]c, blue) showed that there is a decay of the vector components parallel to the effective field in addition to the precession around $\nu_{\mathrm{eff}}$ . This effect is already clearly visible for $t_{p}=10\;µs$ and culminates in M(t→∞)=0 for even longer pulses (not shown). In agreement with the density matrix simulations in the rotating frame, this leads to an increased hole depth for a certain offset $\Omega_{S}$ and, thus, to a broadening of the burned hole. Quantitative analysis of the width of the calculated hole profiles by their full width at half maximum (FWHM) value (Fig. [Fig Ch1.F8]d) showed that, while the tilted-frame solution approached a steady state at the theoretical value of $2\nu_{1}$ (horizontal black line), the width of the hole calculated in the rotating frame continuously increased. This effect becomes significant for $t_{p}≳T_{2\rho }$ (vertical black line). The experimental FWHM (from the data in Fig. [Fig Ch1.F5]) shows only a slight increase in the FWHM for long $t_{p}$ that is not consistent with the rotating-frame results. We attribute the comparably small increase in the FWHM to spectral diffusion as observed, for example, by [Bibr bib1.bibx31].

## 5 Conclusions

In this work, we demonstrated the feasibility of shaped-pulse experiments using periodic phase modulations and chirp pulses on a commercial Bruker E680 W-band spectrometer. These experimental tools were then applied to the analysis of electron spin dynamics during spin locking. In particular, we reported that spin dynamics during MW irradiation obey principles similar to the bare state, i.e., Larmor, precession around effective fields and transverse relaxation in the eigenframe of the complete spin Hamiltonian. We performed tilted-frame experiments to measure SL relaxation times and to analyze the relation between bare and SL dynamics. Inversion and excitation profiles of low-power MW pulses were used to benchmark density matrix simulations of these SL dynamics in a reduced spin system. Quantitative agreement was reached using experimental parameters without modification. Additional Spinach-based simulations, including electron–nuclear coupling, powder averaging, and explicit detection, suggested that our findings can be generalized to more complex spin systems and simulation routines. Comparison of these results with simulations based on the Bloch equations showed that, in both cases, transformation into the tilted eigenframe of the MW Hamiltonian is needed for pulse lengths exceeding the SL decoherence time $T_{2\rho }$ to achieve quantitative agreement with the experimental data.

While we performed the experiments with a BDPA radical which has a narrow and unstructured EPR spectrum, we expect some of the results to be transferable to other samples, including the frequently used nitroxide radicals. First chirp EPR experiments of nitroxide radicals are a promising starting point for using FT EPR for broadband experiments on our W-band spectrometer, and the work in this direction is still ongoing.

Multiple further questions still need to be addressed. Knowledge of the dependence of $T_{2\rho }$ on the chemical environment could increase mechanistic understanding of this relaxation process and, ultimately, enable tuning of the relaxation properties in the SL state to increase the decoherence time in pulsed EPR experiments. Furthermore, the effect of $T_{1\rho }$ was not analyzed. A similar analysis of the longitudinal SL relaxation would be interesting if an appropriate benchmark experiment could be designed. Additionally, a detailed, quantitative analysis of the hyperfine decoupling in the SL state could be used to estimate the applicability of hyperfine spectroscopic experiments in the tilted frame.

## Figures and Tables

**Figure A1:**
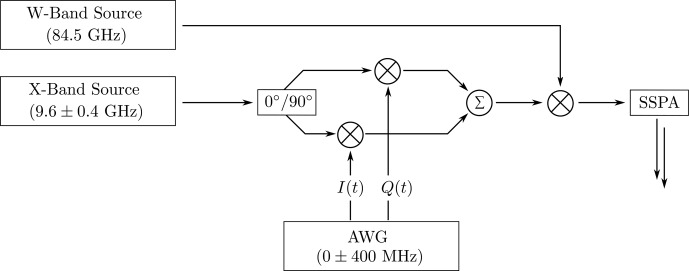
Schematic description of the pulse generation in a Bruker E680 W-band EPR spectrometer equipped with an AWG. See Sect. [Sec Ch1.S3.SS2] for more details.

**Figure A2 App1.Ch1.S1.F10:**
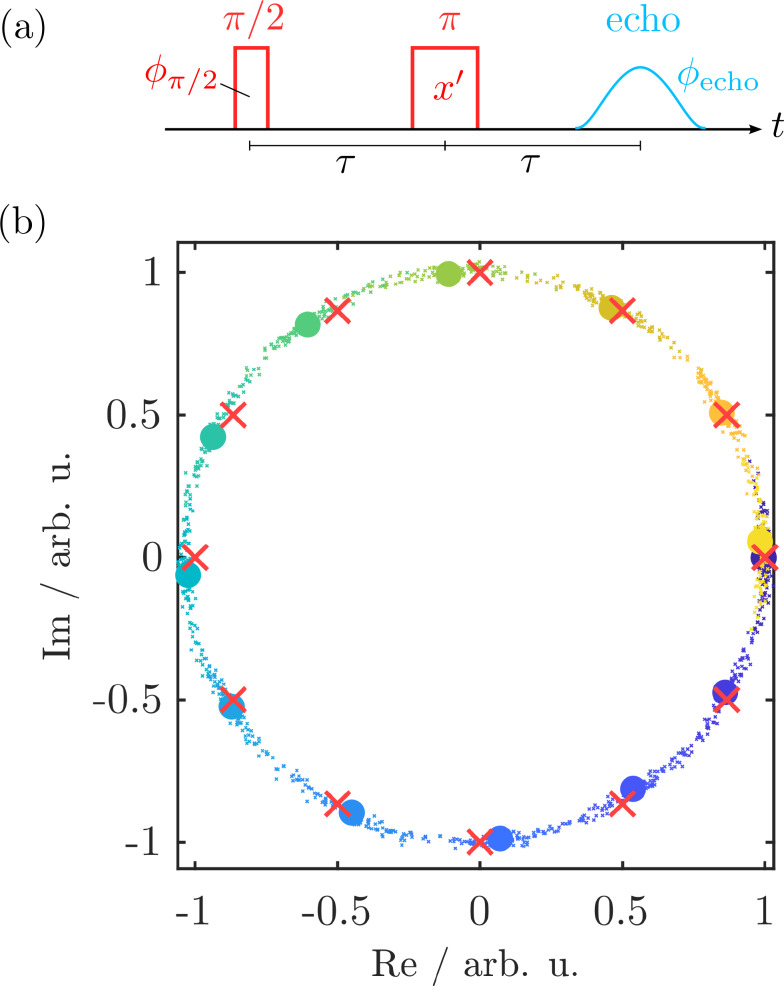
**(a)** Pulse sequence to measure the phase setting and stability using the AWG. The phase of the rectangular π/2 pulse was varied, while the π pulse was kept with a constant phase. **(b)** Real and imaginary contributions of the echo transients obtained with the pulse sequence in **(a)**. The small dots mark a hundred individual scans for each phase $\phi_{\pi /2}$ belonging to one color. The average of these results is marked by a larger filled circle of the respective color. The red “x” symbols mark the theoretical position. Experimental parameters: $t_{\pi }=60\;\mathrm{ns}$ , and τ=1µs .

**Figure A3 App1.Ch1.S1.F11:**
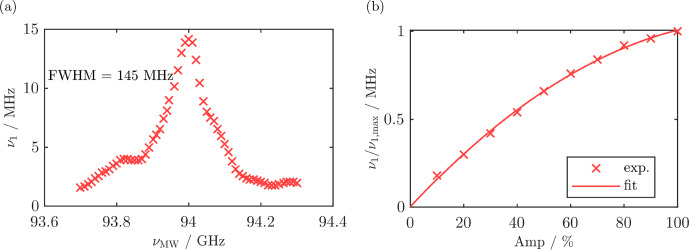
**(a)** Resonator profile used for the correction of the chirp pulses. Acquisition was done at 50K by measuring two-pulse Rabi nutations of BDPA at different local oscillator frequencies. **(b)** Amplification curve of the solid-state amplifier used for correction of the chirp pulses. The data were obtained by room temperature measurements of BDPA Rabi nutations at different attenuation values of the AWG output voltage. The latter are given as relative *amplitude* (Amp) values that can be between 0% and 100% . A second-order polynomial $f(x)=a+bx+cx^{2}$ was used for fitting. Pulse sequence: pulse-T-π/2-τ-π-τ-echo . Experimental parameters: $t_{\pi }\approx 40$ – 400ns , T≈10µs , and τ≈0.3 – 1µs .

**Figure A4 App1.Ch1.S1.F12:**
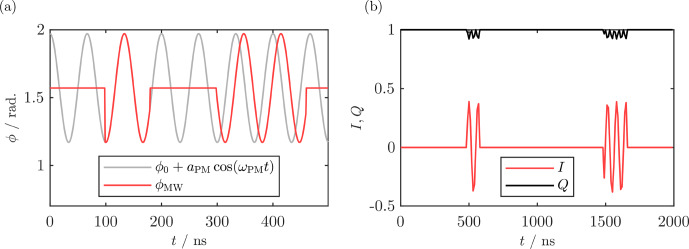
**(a)** MW phase $\phi_{\mathrm{MW}}$ during a representative pulse shape including PM pulses (red) and corresponding carrier wave after. The following parameters were used: $\nu_{1}=\nu_{\mathrm{PM}}=15\;\mathrm{MHz}$ , $\phi_{0}=\pi /2$ , $a_{\mathrm{PM}}=0.4$ , $\tau_{0}=100\;\mathrm{ns}$ , $\tau_{1}=\tau_{2}=200\;\mathrm{ns}$ , $t_{\mathrm{PM}1}=80\;\mathrm{ns}$ , $t_{\mathrm{PM}2}=160\;\mathrm{ns}$ , $\phi_{\mathrm{PM}1}=0$ , and $\phi_{\mathrm{PM}2}=\pi /2$ . A time increment of 1ns was used for better visualization. Note the shift of PM2 with respect to the carrier wave due to its PM phase. **(b)** I(t) and Q(t) values of a representative pulse shape including PM pulses as uploaded to the spectrometer. The following parameters were used: $\nu_{1}=\nu_{\mathrm{PM}}=15\;\mathrm{MHz}$ , $\phi_{0}=\pi /2$ , $a_{\mathrm{PM}}=0.4$ , $\tau_{0}=\tau_{2}=500\;\mathrm{ns}$ , $\tau_{1}=1000\;\mathrm{ns}$ , $t_{\mathrm{PM}1}=80\;\mathrm{ns}$ , $t_{\mathrm{PM}2}=160\;\mathrm{ns}$ , $\phi_{\mathrm{PM}1}=0$ , and $\phi_{\mathrm{PM}2}=\pi /2$ . A time increment of 10ns was used in agreement with the experiments.

**Figure A5 App1.Ch1.S1.F13:**
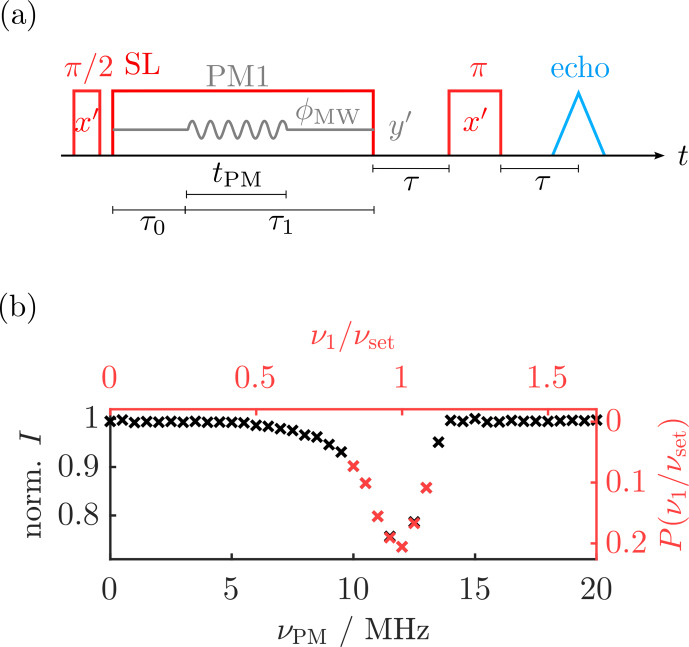
**(a)** Pulse sequence for measuring the frequency-stepped SL resonance spectrum and result for $\nu_{\mathrm{set}}=12$ MHz (**b**, black). The processed data used for simulations are shown in red. Experimental parameters: $t_{\pi }=48\;\mathrm{ns}$ , $\tau_{0}=1\;µs$ , $\tau_{1}=2\;µs$ , $t_{\mathrm{PM}}=1\;µs$ , $a_{\mathrm{PM}}=0.04$ , τ=0.3µs , SPP=5 , and four-step PC of the π detection pulse.

**Figure A6 App1.Ch1.S1.F14:**
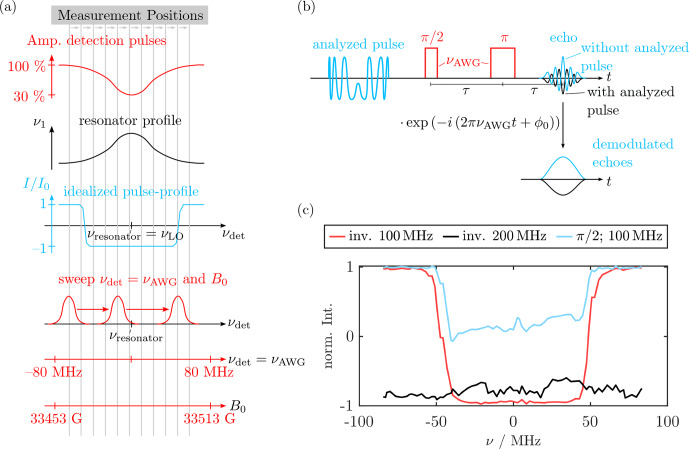
**(a)** Experiment used for acquisition of inversion profiles together with the used pulse sequence **(b)**, following the procedure of [Bibr bib1.bibx17]. **(c)** Representative inversion profiles of 1µs WURST chirp pulses corrected for the resonator profile. Inversion (inv.) pulses were performed with maximal $\nu_{1}$ , while the $\nu_{1}$ value of the π/2 pulse was optimized with a chirp nutation (see Fig. [Fig App1.Ch1.S1.F15]). The frequency value in the legend gives the sweep range the pulses were optimized for. Experimental parameters: $t_{\pi }=72\;\mathrm{ns}$ , τ=0.6µs , and SPP=5 .

**Figure A7 App1.Ch1.S1.F15:**
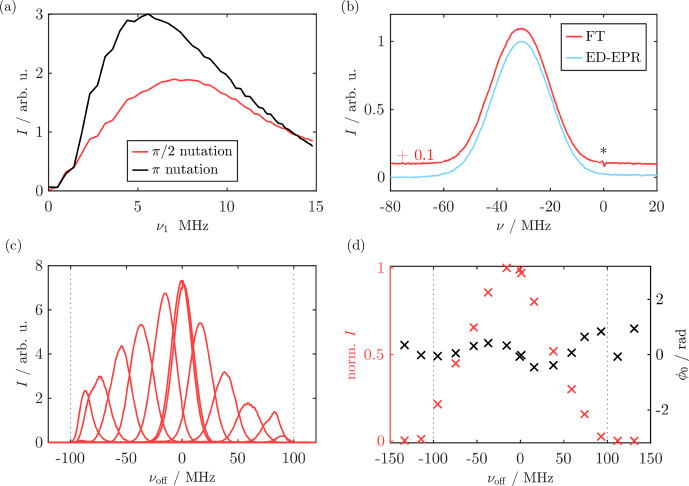
**(a)** Representative chirp echo nutations for the π/2 and π chirp pulses using a 1µs and 0.5µs chirp echo with a sweep width of 200MHz . The π/2 nutation was performed with $\nu_{1,\pi }=10\;\mathrm{MHz}$ , while the π nutation was acquired using the optimized value of $\nu_{1,\pi /2}=7.7\;\mathrm{MHz}$ . **(b)** Comparison of the BDPA EPR spectrum obtained by FT of a chirp echo (red) and by echo-detected EPR (light blue). The asterisks denotes a zero-frequency artifact in the FT. **(c)** FT EPR spectra of BDPA as a function of the frequency offset is shown. Acquisition was done by variation of the magnetic field. **(d)** Integral of each FT spectrum is shown as a function of the frequency offset (red) as well as its corresponding phase (black). The dashed lines mark the sweep range of the chirp pulses. Note that the center of the integral profile in **(d)** is shifted to negative $\nu_{\mathrm{off}}$ values by approximately 10MHz , which was presumably caused by tuning imperfections. Pulse sequence: $\pi /2_{\mathrm{chirp}}-\tau -\pi_{\mathrm{chirp}}-\tau -\mathrm{echo}$ . Experimental parameters: $t_{\pi /2}=1$ , $t_{\pi }=0.5$ , τ≈1-2µs , SPP≈20 , and eight-step PC.

**Figure A8 App1.Ch1.S1.F16:**
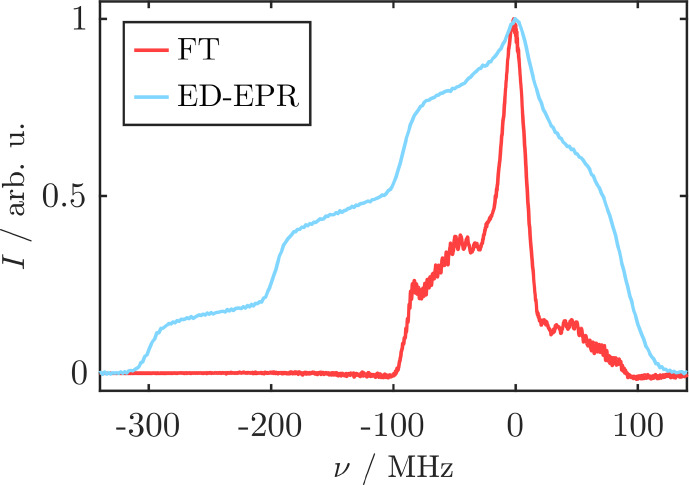
Comparison of the TEMPOL EPR spectrum obtained by FT of a chirp echo (red) and by echo-detected EPR (light blue). For the latter, the field axis was converted into the corresponding frequency axis and horizontally shifted to overlap with the FT spectrum. FT pulse sequence: $\pi /2_{\mathrm{chirp}}-\tau -\pi_{\mathrm{chirp}}-\tau -\mathrm{echo}$ . Experimental parameters: $t_{\pi /2}=1$ , $t_{\pi }=0.5$ , τ=2µs , SPP=2000 , and eight-step PC.

**Figure A9 App1.Ch1.S1.F17:**
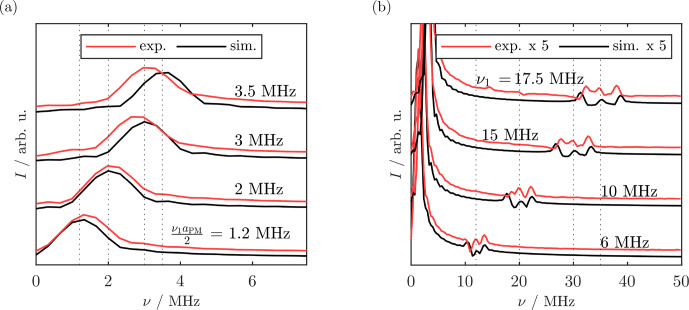
FT spectra of the experimental SL Rabi nutation traces (red) obtained with a fixed $a_{\mathrm{PM}}=0.4$ and different $\nu_{1}$ values. **(a)** Low-frequency region on the order of the SL Rabi frequency $\frac{a_{\mathrm{PM}}\nu_{1}}{2}$ . **(b)** Complete frequency region. FTs of simulated SL Rabi nutations with the same MW power are displayed in black. Vertical lines mark the positions where a signal is expected, i.e., at $\nu =\frac{a_{\mathrm{PM}}\nu_{1}}{2}$ in **(a)** and at $\nu =2\nu_{1}$ in **(b)**. For experimental parameters, see Sect. [Sec App1.Ch1.S2.SS1].

**Figure A10 App1.Ch1.S1.F18:**
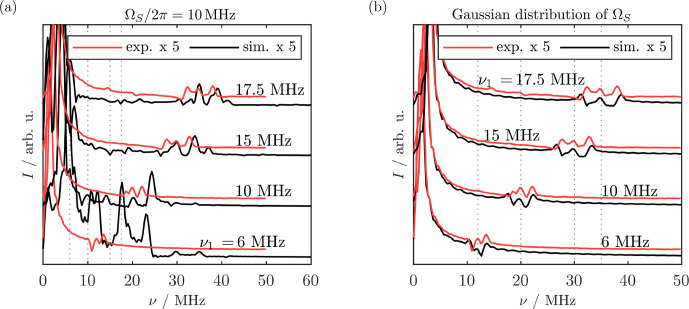
FTs of SL Rabi nutations (red, as in Fig. [Fig App1.Ch1.S1.F17]) compared to simulations with a single electron spin offset $\Omega_{S}/2\pi =10\;\mathrm{MHz}$ **(a)** and with a Gaussian distribution of $\Omega_{1}$ values **(b)**. For the latter, the weighted sum of 2001 traces calculated for $\Omega_{S}/2\pi$ between -24 and 24MHz was calculated using weighting factors from a Gaussian distribution function centered at 0MHz and with FWHM=24MHz .

**Figure A11 App1.Ch1.S1.F19:**
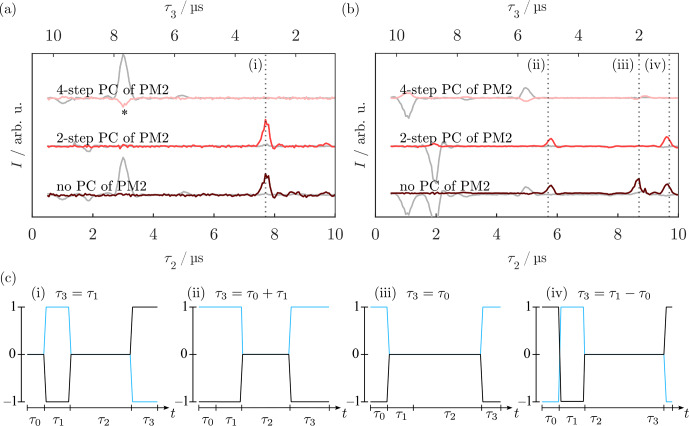
**(a, b)** Imaginary signal (red) of the SL echo experiments shown in Fig. [Fig Ch1.F3]c and d for the SL echo and WOP SL echo, respectively. For comparison, the real parts of the signals (see Fig. [Fig Ch1.F3]) are shown in gray. The asterisk marks an artifact arising from phase drifts between the experiment. **(c)** Coherence diagrams explaining the signals (i) to (iv). All spectra were obtained with an additional two-step PC on PM3. Thus, only signals where PM3 acted as a $\frac{\pi }{2}$ pulse are visible. For experimental parameters, see Sect. [Sec App1.Ch1.S2.SS2].

**Figure A12 App1.Ch1.S1.F20:**
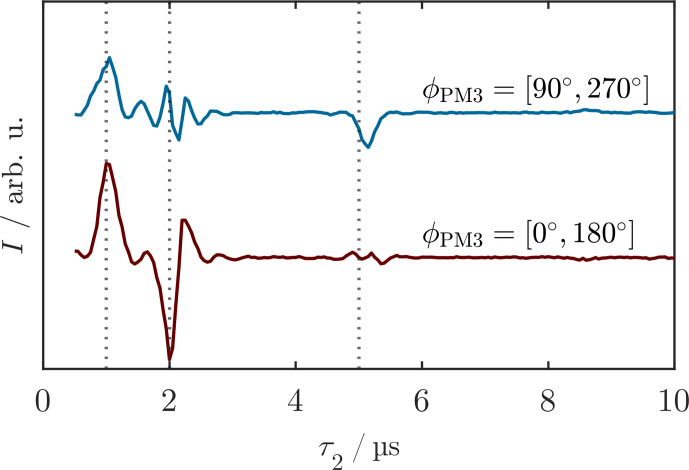
WOP SL echo measured with standard (dark red) and 90° phase-shifted (blue) PM3 pulse. The former is obtained under identical conditions as the bottom trace in Fig. [Fig Ch1.F3]d. For experimental parameters, see Sect. [Sec App1.Ch1.S2.SS2].

**Figure A13 App1.Ch1.S1.F21:**
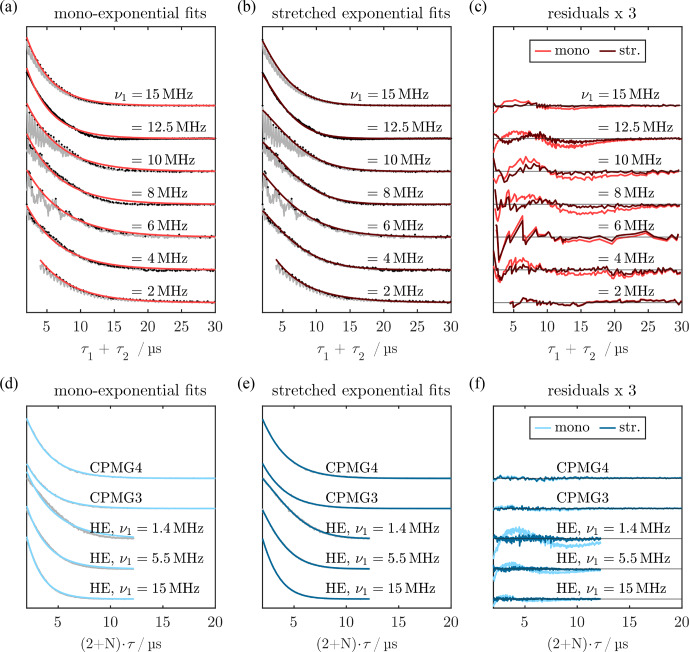
Experimental SL echo decay traces (gray) and the corresponding mono-exponential **(a)** and stretched-exponential **(b)** fits. Data points selected for the envelope fitting are displayed as black dots. The corresponding $T_{2\rho }$ values can be found in Fig. [Fig Ch1.F4]. **(c)** Residuals of both fits, scaled by a factor of 3 for better visualization. **(d–f)** Analogous data for the Hahn echo and the Carr–Purcell decay measurements, where N=0 for Hahn echo decays and N=1,2 for CP3 and CP4, respectively. The corresponding $T_{m}$ values can be found in Figs. [Fig Ch1.F4] and [Fig App1.Ch1.S1.F23]. For experimental parameters, see Sect. [Sec App1.Ch1.S2.SS3].

**Figure A14 App1.Ch1.S1.F22:**
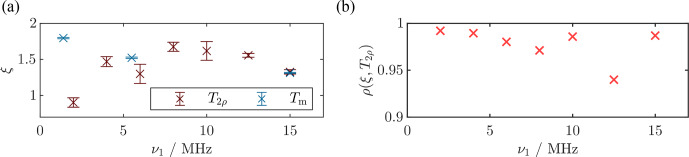
**(a)** Stretching exponents ξ for the $T_{2\rho }$ fits (dark red) and for $T_{m}$ (dark blue) in Fig. [Fig App1.Ch1.S1.F21]b and e. **(b)** Pearson correlation coefficient for ξ and $T_{2\rho }$ of the different fits in Fig. [Fig App1.Ch1.S1.F21]b and e calculated from the fitting results of the bootstrap samples. For experimental parameters, see Sect. [Sec App1.Ch1.S2.SS3].

**Figure A15 App1.Ch1.S1.F23:**
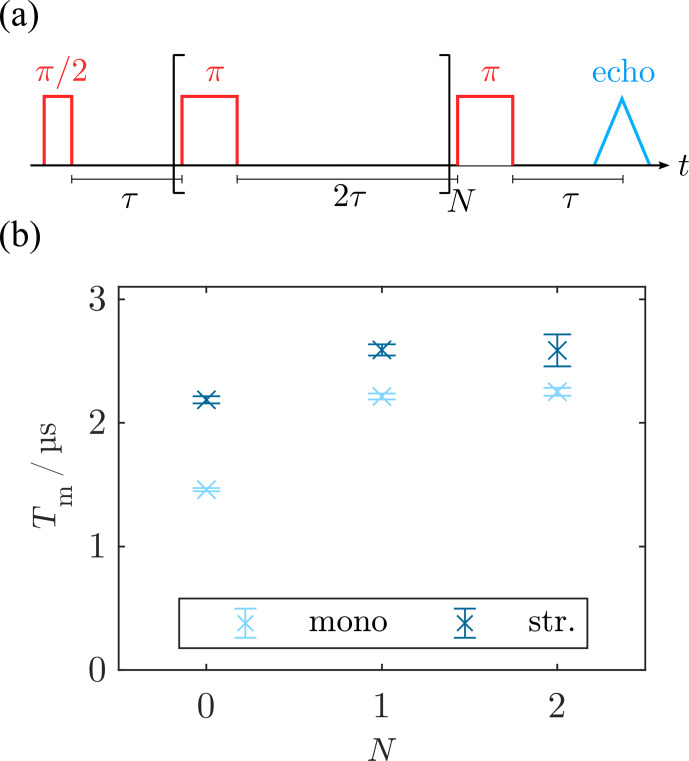
**(a)** Pulse sequence for the Carr–Purcell (CP) experiment with N repetitions of the central refocusing block. **(b)** Decoherence times were obtained from the fitting in Fig. [Fig App1.Ch1.S1.F21]d–f for the Hahn echo decay ( N=0 ) and the CP3 and CP4 experiments ( N=1,2 , respectively). For N=1,2 , a 16-step PC of the last two π pulses was applied. All experiments were performed with $\nu_{1}=15\;\mathrm{MHz}$ . For further experimental parameters, see Sect. [Sec App1.Ch1.S2.SS3].

**Figure A16 App1.Ch1.S1.F24:**
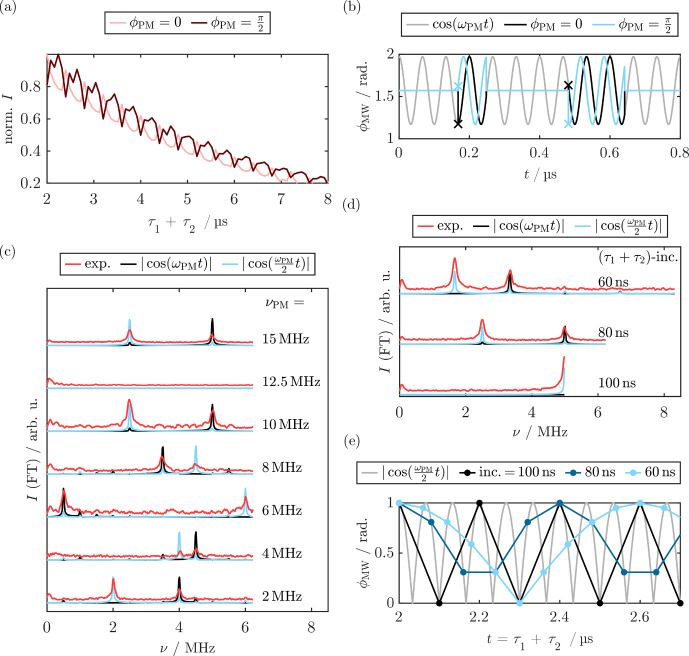
**(a)** Excerpt of $T_{2\rho }$ nutation traces obtained with $\nu_{1}=15\;\mathrm{MHz}$ and $\phi_{\mathrm{PM}}=0,\frac{\pi }{2}$ . **(b)** Schematic drawing of arbitrary PM pulses for $\phi_{\mathrm{PM}}=0,\frac{\pi }{2}$ (black, blue). The first points of the PM pulses are marked with an “x ” symbol and a continuous cosine wave as a reference for $\phi_{\mathrm{PM}}=0$ is marked in gray. FTs of the experimental $T_{2\rho }$ traces for different MW powers $\nu_{1}$ **(c)** and increments of $t=\tau_{1}+\tau_{2}$ **(d)** show distinct frequencies (red). Assuming undersampling of oscillations $\left|\mathrm{cos}(\omega_{\mathrm{PM}}t)\right|$ and $\left|\mathrm{cos}\left(\frac{\omega_{1}}{2}t\right)\right|$ , the FT of the resulting traces yielded alias frequencies in quantitative agreement with the oscillations observed in the experiment (blue and black in **c** and **d**). **(e)** Illustration of alias frequencies for a $\left|\mathrm{cos}\left(\frac{\omega_{1}}{2}t\right)\right|$ oscillation with $\nu_{\mathrm{PM}}=15\;\mathrm{MHz}$ and different sampling increments as used in **(d)**. For experimental parameters, see Sect. [Sec App1.Ch1.S2.SS3].

**Figure A17 App1.Ch1.S1.F25:**
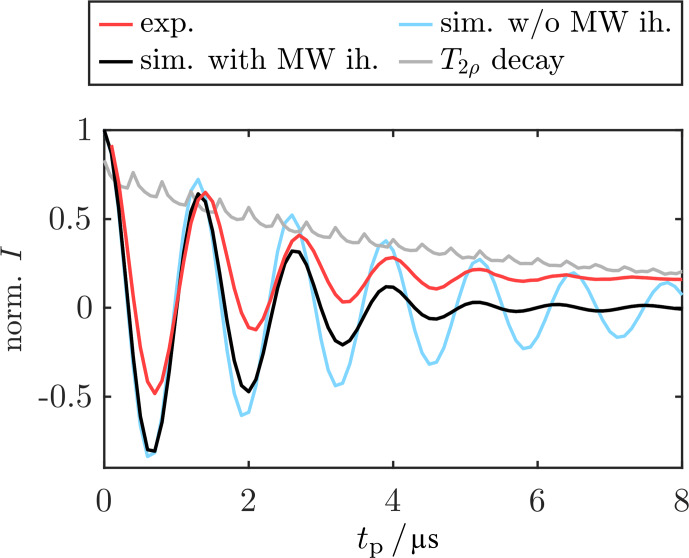
Experimental Rabi nutation obtained from the CHEESY z profiles (red) and comparison with the simulation with (black) and without (blue) including MW inhomogeneity (ih.) using the $\nu_{1}$ distribution function $P(\nu_{1})$ . For both simulations, $T_{2\rho }$ was set to 4µs . For comparison, the experimental $T_{2\rho }$ decay ( $\nu_{\mathrm{PM}}=15$ MHz) is shown in gray. For experimental parameters, see Sect. [Sec App1.Ch1.S2.SS4].

**Figure A18 App1.Ch1.S1.F26:**
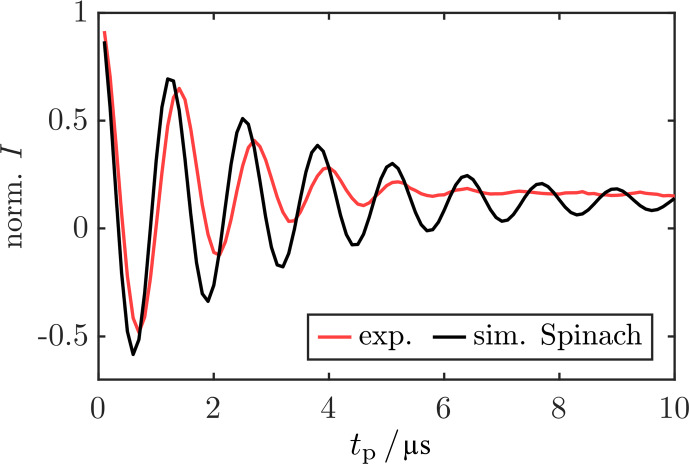
Experimental Rabi nutation obtained from the CHEESY z profiles (red) and comparison with the Spinach simulation without MW inhomogeneity (black). The simulation results closely match the simulation results with the home-written density matrix approach without MW inhomogeneity (light blue curve in Fig. [Fig App1.Ch1.S1.F25]). For experimental parameters, see Sect. [Sec App1.Ch1.S2.SS4].

**Figure A19 App1.Ch1.S1.F27:**
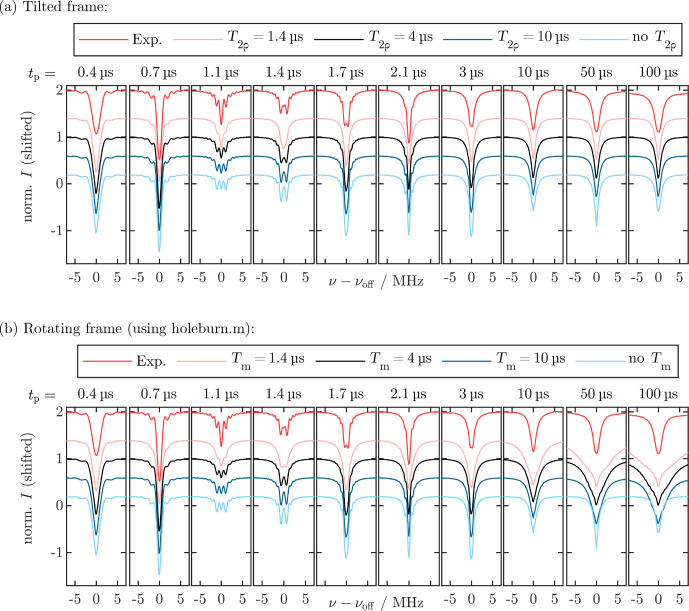
**(a)** Experimental CHEESY $z^{\prime }$ profiles (red) compared to results from Spinach simulations in the tilted frame with different values of $T_{2\rho }$ . The spectra were shifted vertically for the sake of clarity. **(b)** Analogous simulations performed in the rotating frame using the holeburn.m function of Spinach together with the t1_t2.m model for relaxation.

**Figure A20 App1.Ch1.S1.F28:**
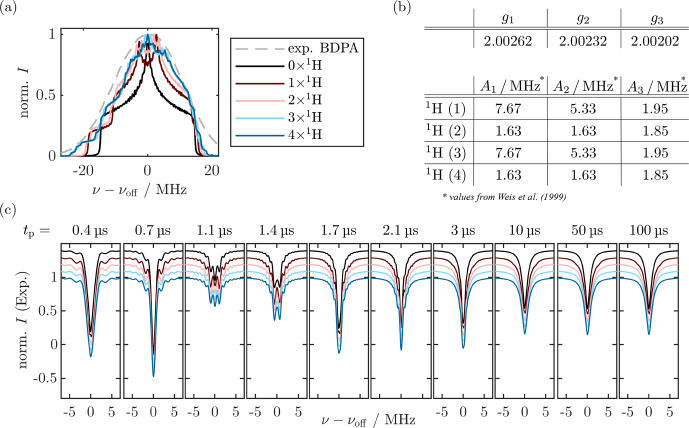
**(a)** Normalized EPR spectra of an electron spin with g anisotropy coupled to up to four proton nuclei simulated using the Spinach library [Bibr bib1.bibx29]. The experimental FT spectrum of BDPA (see Fig. [Fig App1.Ch1.S1.F15]b) is displayed as a dashed gray line. **(b)** Parameters used for the simulations. Elements of the g tensor were set to arbitrary values to achieve sufficient spectral width. Hyperfine couplings are taken from [Bibr bib1.bibx64] for ^2^H-BDPA and corrected by the ratio of the gyromagnetic ratios of ^1^H and ^2^H. Protons one to four were added successively to obtain the spectra with zero to four coupled nuclei. **(c)** CHEESY profiles for the spin systems with zero to four nuclei (same color code as in **a**) simulated with the Spinach library. For clarity, the spectra are vertically shifted.

## Data Availability

All experimental data, data analysis scripts, and simulation code are available for download from the Göttingen Research Open Data Repository under 10.25625/B11CUC [Bibr bib1.bibx43].

## Appendix A Supplementary data and analysis

### A2 Phase stability of the spectrometer

To assess the phase stability of the pulses produced by the AWG, we performed a spin echo experiment with transient detection as described by [Bibr bib1.bibx21]. The results are displayed in Fig. [Fig App1.Ch1.S1.F10]. Analysis of the data shows that strong phase noise is observable, likely due to a deteriorating performance of the 84.5GHz MW source. When looking at the average of a hundred shots (filled circles), the phase agrees reasonably well with the theoretical value (red “x” symbols), suggesting that phase cycling is possible whenever large numbers of scans are used. This is in agreement with our experiments where we observe efficient phase cycling. However, this procedure will lead to a loss in the signal-to-noise ratio due to partial cancellation.

### A3 Resonator profile and amplifier characterization

Figure [Fig App1.Ch1.S1.F11]a shows the $\nu_{1}$ profile of the Bruker EN600-1021H ENDOR resonator measured with BDPA at a temperature of 50K . The profile is clearly asymmetric and shows some oscillations, which are indicative of reflections in the resonator [Bibr bib1.bibx17].

Additionally, Rabi nutation experiments were performed at different AWG output voltages to measure the amplification curve of the SSPA. This was achieved by variation of the *amplitude* parameter (Amp) in the Xepr software that scales the input IQ voltage values by a factor between 0% and 100% . Due to saturation effects, the amplification curve was clearly nonlinear. Therefore, all nominal MW powers were corrected with this curve in order to yield the target $\nu_{1}$ .

### A4 Generation of PM pulses

PM pulses were generated with a home-written MATLAB routine following Eq. (5) [Bibr bib1.bibx67]. Figure [Fig App1.Ch1.S1.F12]a shows two PM pulses with phases of $\phi_{\mathrm{PM}1}=0$ and $\phi_{\mathrm{PM}2}=\pi /2$ , respectively. The I(t) and Q(t) values are calculated from $\phi_{\mathrm{MW}}$ using Eq. ([Disp-formula App1.Ch1.S1.E18]):

A1
$$I(t)=\mathrm{cos}(\phi_{\mathrm{MW}}),\;Q(t)=\mathrm{sin}(\phi_{\mathrm{MW}}).$$

A representative pulse shape including two PM pulses is shown in Fig. [Fig App1.Ch1.S1.F12]b. Note that some distortions caused by the comparably large time increment of 10ns are visible for $\nu_{\mathrm{PM}}=15\;\mathrm{MHz}$ . However, choosing a smaller time increment was impossible due to the limited number of IQ values for a single pulse shape supported by the Xepr software.

### A5 Calibration of $\nu_{\mathrm{PM}}$ and measurement of the $\nu_{1}$ profile

The frequency-stepped SL resonance spectrum was measured to set $\nu_{\mathrm{PM}}=\nu_{1}$ [Bibr bib1.bibx67]. In this experiment, a single long PM pulse with low modulation amplitude ( $t_{\mathrm{PM}}=1\;µs$ and $a_{\mathrm{PM}}=0.04$ ) was applied during SL (Fig. [Fig App1.Ch1.S1.F13]a). This caused a signal decrease proportional to the degree of matching of $\nu_{1}$ and $\nu_{\mathrm{PM}}$ . The frequency $\nu_{\mathrm{PM}}$ with a maximal signal decrease was used in the PM pulse experiments (i.e., $\nu_{\mathrm{set}}$ ; see Fig. [Fig App1.Ch1.S1.F13]b, black).

To obtain a discrete probability distribution function of $\nu_{1}$ (i.e., $P(\nu_{1})$ ) from this experiment, the spectrum was vertically shifted and normalized. The abscissa was scaled by $\nu_{\mathrm{set}}$ to allow for transfer between experiments with different $\nu_{\mathrm{set}}$ values. For simulation of MW inhomogeneity, all data points exceeding $0.3P(\nu_{1}/\nu_{\mathrm{set}}=1)$ were selected, and the spectrum was again normalized to a sum of one (Fig. [Fig App1.Ch1.S1.F13]b, red). Calculations were repeated for each of these $\nu_{1}/\nu_{\mathrm{set}}$ values and the results weighted by $P(\nu_{1}/\nu_{\mathrm{set}})$ . For the simulation of SL echoes, linear interpolation was used between the selected data points to increase the resolution of the $\nu_{1}/\nu_{\mathrm{set}}$ axis.

### A6 Generation of chirp pulses

Shapes of chirp pulses were generated using a home-written MATLAB routine. After defining the pulse length $t_{p}$ and sweep width $\nu_{\mathrm{SW}}$ , an incremented frequency array of $\nu_{\mathrm{AWG}}$ was generated that spanned the whole sweep range. For each increment i , the length Δt(i) was adapted using the experimental resonator profile in Fig. [Fig App1.Ch1.S1.F11]a so that the critical adiabaticity $Q_{\mathrm{crit}}(i)$ (Eq. A2) was constant throughout the pulse and equal to its mean value $\bar{Q_{\mathrm{crit}}}$ [Bibr bib1.bibx3].

A2Qcrit(i)=2π[ν1(i)]2Δtp(i)Δν=Qcrit‾,A3Qcrit‾=2πν12‾tpνSW.

Here, $\nu_{1}(i)$ is the Rabi frequency for increment i as obtained from the resonator profile. Δν corresponds to the constant frequency increment $\nu_{\mathrm{AWG}}(i+1)-\nu_{\mathrm{AWG}}(i)$ . $\Delta t_{p}$ is the length of time increment i , and $\bar{\nu_{1}^{2}}$ is the mean value of $\nu_{1}^{2}$ as obtained from the resonator profile.

The nonlinear frequency sweep was interpolated to fit the constant AWG sampling rate of $\Delta t_{\mathrm{AWG}}=0.625\;\mathrm{ns}$ and converted to a phase modulation by Eq. ([Disp-formula App1.Ch1.S1.E21]) [Bibr bib1.bibx18].

A4
$$\phi_{\mathrm{AWG}}(t)=\int_{0}^{t}2\pi \nu_{\mathrm{AWG}}(t^{\prime })dt^{\prime }+\phi_{\mathrm{AWG}}(0)$$

This phase was inserted into Eq. ([Disp-formula App1.Ch1.S1.E18]), yielding the AWG input I(t) and Q(t) . A wideband, uniform rate, smooth truncation (WURST) amplitude modulation function was applied to these I(t) and Q(t) values in order to reduce pulse edge artifacts [Bibr bib1.bibx39]:

A5
$$\begin{array}{rl} I_{\mathrm{WURST}}(t)= & I(t)\cdot \left(1-{\left|\mathrm{cos}\left(\frac{\pi t}{t_{p}}\right)\right|}^{n_{W}}\right)\\ & \mathrm{where}\;\;t\in [0,t_{p}], \end{array}$$

where $n_{W}$ is a parameter that reflects the steepness of the WURST modulation function. For the experiments shown in this work, $n_{W}=50$ was used. The final pulse shapes were obtained after correction with the amplifier profile (see Fig. [Fig App1.Ch1.S1.F11]b). In order to assess the performance of these pulses, we measured inversion profiles of representative pulse shapes. We used a spin echo experiment as illustrated in Fig. [Fig App1.Ch1.S1.F14]a, b and compared the echo intensity with and without the pulse of interest [Bibr bib1.bibx17]. In contrast to the work by [Bibr bib1.bibx2], we varied the frequency of the detection sequence and the $B_{0}$ field, while keeping the analyzed pulse at a constant frequency centered at the resonator dip with $\nu_{\mathrm{resonator}}=\nu_{\mathrm{LO}}$ . In this way, the shaped pulses stayed fixed with respect to the resonator profile, and we could monitor the effect of the resonator bandwidth on the inversion properties of the pulses. In the case where the detection frequency is fixed at the position of the resonator dip, we would expect overestimation of the pulse performance because inversion is always measured at the position with maximal $\nu_{1}$ .

With this procedure, two factors had to be taken into account. First, $\nu_{1}$ of the detection pulses depends on their position with respect to the resonator profile. In order to keep the pulse length constant, the AWG output voltage (set by the *amplitude* parameter in the Xepr software) of the detection pulses was scaled with the inverse resonator profile [Bibr bib1.bibx17]. Second, signal detection by the spectrometer is done in a quadrature detection scheme by demodulation with $\nu_{\mathrm{PLO}}$ and $\nu_{x}$ . Thus, the resulting echo was oscillating with $\nu_{\mathrm{AWG}}$ that was different for each data point. We demodulated the echoes using a complex exponential function oscillating with $\nu_{\mathrm{AWG}}$ (see Fig. [Fig App1.Ch1.S1.F14]b). $\phi_{0}$ was optimized for the background spectrum without the analyzed pulse and then used for the echo with pulse without further modification. Figure [Fig App1.Ch1.S1.F14]c shows the inversion profiles of representative chirp pulses with a pulse length of $t_{p}=1\;µs$ in a frequency range of ±80MHz . While the π pulse with $\nu_{\mathrm{SW}}=100\;\mathrm{MHz}$ shows good inversion efficiency over the whole sweep range, some artifacts are visible in the case of $\nu_{\mathrm{SW}}=200\;\mathrm{MHz}$ . The pulse with $\nu_{1}$ optimized for a π/2 pulse shows a reasonably flat profile with $I/I_{0}\approx 0$ as expected.

### A7 Characterization of chirp echoes

Figure [Fig App1.Ch1.S1.F15]a shows the chirp nutations to determine the ideal $\nu_{1}$ value for both the π/2 and the π chirp pulse [Bibr bib1.bibx17]. Despite the low $Q_{\mathrm{crit}}$ of the chirp π pulse (see Sect. [Sec Ch1.S3.SS5]), the FT of a chirp echo of BDPA showed quantitative agreement with the corresponding ED EPR spectrum, as it is shown in Fig. [Fig App1.Ch1.S1.F15]b. For the latter, the field axis B was converted into a frequency axis ν using $\nu =\nu_{\mathrm{off},\mathrm{FS}}-g_{e}\mu_{B}hB$ ; $\nu_{\mathrm{off},\mathrm{FS}}$ was selected manually to align the ED EPR with the FT spectrum. Figure [Fig App1.Ch1.S1.F15]c shows the phased chirp echo FT spectrum of BDPA as a function of the offset relative to the center of the resonator profile [Bibr bib1.bibx21]. The corresponding integral and the phase $\phi_{0}$ of the signal are shown in (d). The intensity profile closely follows the resonator profile. Fig. [Fig App1.Ch1.S1.F16] shows the analogous comparison for a TEMPOL radical. The spectral width exceeds the sweep range of the chirp pulses by more than 200MHz , which prevented the detection of the full EPR spectrum by FT spectroscopy. Within the sweep range, the FT spectrum is the approximate convolution of the echo detected EPR and the resonator profile. Additionally, there are some further distortions in the line shape, presumably caused by the pulse edges or reflections in the MW resonator. Although this result shows the limitations of our experimental setup to completely detect broad EPR spectra, it suggests that FT EPR might be useful for other applications to partially benefit from the multiplex advantage of the FT approach.

### A8 SL Rabi nutations

The signals observed in the FT of Fig. [Fig Ch1.F2], centered at the SL Rabi frequency $\frac{a_{\mathrm{PM}}\nu_{1}}{2}$ and at $2\nu_{1}$ , were also retained when varying $\nu_{1}=\nu_{\mathrm{PM}}$ (see Fig. [Fig App1.Ch1.S1.F17]). Again, simulations agreed well with the experimental data and the theoretical signals.

If a single, bare-spin offset $\Omega_{S}$ was introduced to the simulation, a strong distortion of the FT spectra occurred for $\Omega_{1}≳\omega_{1}$ (Fig. [Fig App1.Ch1.S1.F18]a). In contrast, simulation of a Gaussian distribution of $\Omega_{S}$ values with FWHM=24MHz did not yield these artifacts (Fig. [Fig App1.Ch1.S1.F18]b).

### A9 Phase of SL echo experiments

During a SL echo experiment, we can distinguish between two kinds of phase information. On the one hand, the final integrated echo has a phase depending on the magnetization phase at the end of the SL pulse. On the other hand, the SL echo itself (i.e., the one generated by the PM pulses) has a phase relative to the readout PM3 pulse. As both of these phases carry additional experimental information, these will be discussed in the following section. The imaginary signal component of the SL echo experiments in Fig. [Fig Ch1.F3], which refers to the phase of the integrated echo, is shown in Fig. [Fig App1.Ch1.S1.F19]. Comparison of real and imaginary component shows that the signals are clearly separated in the $\tau_{2}$ dimension. While the real signals can be assigned to spins that are refocused at the end of $\tau_{2}$ , the imaginary signals all stem from spins refocused at the end of $\tau_{3}$ . Namely, (i), (ii), and (iii) can all be explained by stimulated echoes resulting from different combinations of delays. Signal (iv) is a combination of a refocused echo followed by a stimulated echo. Please note that only signals where PM3 acts as a π/2 pulse are visible because of the two-step PC of PM3 in all experiments. The signal in (a) that is marked with an asterisk is a contribution from the real part caused by phase drifts between the experiments.

This phase separation of the SL echo signals differs from the phase of the SL echoes at the position of PM3. In order to measure this phase, quadrature detection was applied on the readout pulse PM3. Namely, its two-step phase cycle that was applied in all SL echo experiments was shifted by 90° so that, instead of $\phi_{\mathrm{PM}3}=[0{}^{\circ},180{}^{\circ}]$ , $\phi_{\mathrm{PM}3}=[90{}^{\circ},270{}^{\circ}]$ was used. The results for the different phases are displayed in Fig. [Fig App1.Ch1.S1.F20]. Although the experiment was performed under nominally the same conditions as the one shown in Fig. [Fig Ch1.F3]d, the relative phase of the signals with $\phi_{\mathrm{PM}3}=[0{}^{\circ},180{}^{\circ}]$ is different in this spectrum. As the first peak is caused by a refocused echo and the second by a stimulated echo, an inverted sign would be expected for the two, which is the case in Fig. [Fig App1.Ch1.S1.F20] but not in Fig. [Fig Ch1.F3]d. This already shows that the phase of the echoes is not stable in our experiment. Additionally, the imaginary signal of the quadrature detection experiment (i.e., $\phi_{\mathrm{PM}3}=[90{}^{\circ},270{}^{\circ}]$ ) shows significant absorptive contributions for some of the signals. This again demonstrates that the observed phase in this experiment deviates from the theoretically expected one.

### A10 $T_{2\rho }$ measurements

The SL relaxation decays and their mono-exponential and stretched-exponential least-square fits (Eq. A6) corresponding to the $T_{2\rho }$ values in Fig. [Fig Ch1.F4] are shown in Fig. [Fig App1.Ch1.S1.F21]a and b. Equations (A6) and (A7) were used for the fitting:

A6I=I0⋅exp⁡-τ1+τ2T2ρ,A7I=I0⋅exp⁡-τ1+τ2T2ρξ.

Oscillations are visible in the beginning of some of the experimental traces. Following the literature concerned with the fitting of $T_{m}$ traces that include electron spin echo envelope modulation (ESEEM) [Bibr bib1.bibx57], we fitted the envelope function of the oscillation maxima. Specifically, we generated a time window with a length of the inverse of the main oscillation frequency for each data point. The data point with maximal intensity was selected for each of these windows. After removing the redundancies in this selected data set, only the data points at a maximum of the dominant oscillation were obtained. A detailed analysis of the oscillation frequencies can be found in Sect. [Sec App1.Ch1.S1.SS12]. Statistical analysis was performed using the bootstrapping method e.g.,. For each N point data set, $N_{\mathrm{boot}}=1000$ synthetic data sets were generated by randomly taking N points from the data set with replacement and thus, allowing points to be selected multiple times. Each of these bootstrap data sets was fitted and the final result and its error were obtained as the mean and standard deviation of the fit parameters from all synthetic data sets. Errors are given as the 95% confidence intervals (i.e., as 2σ ). Note that this only takes into account statistical errors and cannot give an estimate of systematic errors introduced by the fit model. These can instead be analyzed using the residuals of the fitting (Fig. [Fig App1.Ch1.S1.F21]c). The analogous data for the Hahn echo decay and CPMG experiments can be found in Fig. [Fig App1.Ch1.S1.F21]d–f.

Note that the oscillations in the residuals for small $\tau_{1}+\tau_{2}$ indicate that the removal of oscillating components in the decay traces is incomplete (see Sect. [Sec App1.Ch1.S1.SS12] for more detail). Analysis of the Pearson correlation coefficient for $T_{2\rho }$ and ξ in the bootstrap samples was performed using the corrcoef command in MATLAB (see Fig. [Fig App1.Ch1.S1.F22]b). For all values of $\nu_{1}$ , the Pearson correlation of the fitting parameters $\rho (\xi ,T_{2\rho })$ was above 0.9 , which shows that the parameters are highly correlated.

### A11 Carr–Purcell sequence

We measured decoherence times using the Carr–Purcell sequence with three and four pulses (CP3 and CP4). The results are shown in Fig. [Fig App1.Ch1.S1.F23], and the decay traces can be found in Fig. [Fig App1.Ch1.S1.F21]d–f. For both experiments, the decays were well reproduced both by a mono-exponential fit and a stretched-exponential fit, and an increase in decoherence time was observed compared to the two-pulse Hahn echo experiment.

### A12 Oscillations in $T_{2\rho }$ traces

Distortions of the SL state relaxation traces, consisting of oscillations for small $\tau_{1}+\tau_{2}$ values, were observed in most tilted-frame echo decay experiments. As deuteration of the used BDPA radical showed no effect on the oscillation frequency (data not shown), ESEEM-like modulation caused by residual hyperfine couplings could be excluded. Moreover, a phase shift of the oscillation by π was observed when changing $\phi_{\mathrm{PM}}$ by $\frac{\pi }{2}$ for all three PM pulses simultaneously (see Fig. [Fig App1.Ch1.S1.F24]a), which suggested an effect depending on an oscillation with $\left|\mathrm{cos}(\phi_{\mathrm{PM}})\right|$ . FT of the experimental $T_{2\rho }$ decays for different $\nu_{1}$ and $\tau_{1}+\tau_{2}$ increments (red in Fig. [Fig App1.Ch1.S1.F24]c and d) showed seemingly random but well-defined oscillation frequencies.

Considering the principle of PM pulses (Fig. [Fig App1.Ch1.S1.F24]b; [Bibr bib1.bibx67]), it is apparent that, depending on the time t between the beginning of the spin lock and the PM pulse and on the PM phase $\phi_{\mathrm{PM}}$ , there is a jump in $\phi_{\mathrm{MW}}$ at the beginning and end of the PM pulses (blue and black “x” symbols in Fig. [Fig App1.Ch1.S1.F24]b). Its magnitude depends on $\left|\mathrm{cos}(\omega_{\mathrm{PM}}t+\phi_{\mathrm{PM}})\right|$ . As such abrupt changes in pulses are known to produce artifacts, these were a plausible explanation for the observed deviations from an ideal echo decay. Because the 80ns increment of $\tau_{1}+\tau_{2}$ in our experiments was larger than the Nyquist threshold of half of the inverse PM frequency $\frac{1}{2\nu_{\mathrm{PM}}}\approx 30\;\mathrm{ns}$ , not the $\nu_{\mathrm{PM}}$ oscillation but a low-frequency alias would be detected in this case (see Fig. [Fig App1.Ch1.S1.F24]e). Thus, we simulated the oscillations by selecting values from $\left|\mathrm{cos}(\omega_{1}t)\right|$ with the experimental time increment and subsequently Fourier transformed this undersampled oscillation (black in Fig. [Fig App1.Ch1.S1.F24]c and d). The frequencies obtained from this FT agreed quantitatively with some of the signals observed in the experimental FT spectra of the decay traces. Additional signals could be reproduced when repeating the procedure with a $\left|\mathrm{cos}\left(\frac{\omega_{1}}{2}t\right)\right|$ function (blue in Fig. [Fig App1.Ch1.S1.F24]c and d). This is reasonable because, while the position of PM3 shifted with $\tau_{1}+\tau_{2}$ , the position of PM2 varied with $\frac{\tau_{1}+\tau_{2}}{2}$ . Hence, a combination of the pulse edges caused by both PM2 and PM3 could explain most of the observed signals. Importantly, this method also predicts that there is no oscillation in the trace for $\nu_{1}=12.5\;\mathrm{MHz}$ , which agrees well with the experiment. In the future, we could detect the decay traces with a time increment $\tau_{1}+\tau_{2}$ adjusted so that no oscillations are present in the traces. This might improve the results of the fitting for various $\nu_{1}$ .

### A13 Simulation of MW inhomogeneity

Figure [Fig App1.Ch1.S1.F25] shows the experimental Rabi nutation from the CHEESY $z^{\prime }$ profiles (see Fig. [Fig Ch1.F5]d) in red. Additionally, the simulations with and without MW inhomogeneity (black and blue, respectively) are shown. Comparison with an experimental $T_{2\rho }$ decay (gray) shows that experiment and simulation including the $\nu_{1}$ distribution agree well and decay significantly faster than the $T_{2\rho }$ trace. In contrast, the simulation without MW inhomogeneity decays slower and closely resembles the $T_{2\rho }$ decay. The $\nu_{1}$ distribution for calculation of the black curve is taken from the frequency-stepped tilted-frame resonance spectrum without further modification.

### A14 Spinach simulation of the CHEESY z profiles

We chose an electron spin with a rhombic g tensor (see Fig. [Fig App1.Ch1.S1.F28]b) as a model for our Spinach simulations which differs from the almost isotropic g tensor of the BDPA radical. Setting the spin parameters to the literature values known for BDPA could not reproduce the experimental Gaussian line shape, because this shape is caused by the coupling to a large number of nearly equivalent protons in the BDPA molecule. Calculating the line shape accurately would require including all these nuclei into the simulations, which is computationally unfeasible. Hence, an artificial g anisotropy was introduced to generate a broad enough EPR line to monitor the whole inversion profiles. However, this caused a line shape different from a Gaussian EPR line. Namely, without any coupled nuclei, this leads to a distinct rhombic powder pattern with a singularity at $\nu -\nu_{\mathrm{off}}=0$ (black curve in Fig. [Fig App1.Ch1.S1.F28]a). This caused small artifacts in the CHEESY simulations, as the CHEESY intensity scales with the EPR intensity, leading to profiles slightly too narrow in the center. Simulation of MW inhomogeneity was prevented by the increased computation time of the Spinach simulations. Although the shape of the thus simulated profiles agreed well with the experiments upon visual comparison, the Rabi nutation at $\nu -\nu_{\mathrm{off}}=0$ decayed more slowly in the simulation (see Fig. [Fig App1.Ch1.S1.F26]). As described in Sect. [Sec App1.Ch1.S1.SS13] the decay of the experimental Rabi nutation is a combination of decoherence and MW inhomogeneity, which cannot be reproduced when omitting the MW inhomogeneity.

By repeating the simulations with different $T_{2\rho }$ times, the sensitivity of the simulations with regard to the relaxation time was analyzed (Fig. [Fig App1.Ch1.S1.F27]a). Using the bare-state transverse relaxation time $T_{m}=1.4\;µs$ (light red) did not reproduce the experimental profiles as the decay of the sinc oscillations was overestimated. This is most clearly visible for $t_{p}=1.1$ and 1.4µs where significant features of the experimental profiles are missing. In contrast, the visual difference between $T_{2\rho }=4$ and 10µs is small. Therefore, deriving $T_{2\rho }$ solely from the CHEESY profiles is not easily possible. Importantly, omitting $T_{2\rho }$ completely could not reproduce the experimental data (light blue), because no steady state was reached (compare $t_{p}=10$ , 50 , and 100µs ). The analogous simulations with $T_{m}$ in the rotating frame are shown in Fig. [Fig App1.Ch1.S1.F27]b. While the profiles without relaxation (light blue) agree quantitatively with the tilted-frame results, the profiles with relaxation show broadening for large $t_{p}$ values, which is not observed in the experiment. This deviation increased with decreasing decoherence time. Again, this demonstrates that relaxation is the reason why calculations need to be performed in the tilted frame.

Including nuclei led to distinct changes in the simulated EPR spectra (Fig. [Fig App1.Ch1.S1.F28]a). Namely, coupling to one proton nucleus with the known hyperfine coupling tensor of BDPA ( A tensor taken from [Bibr bib1.bibx65]; see Fig. [Fig App1.Ch1.S1.F28]b) led to a distinct splitting (dark red curve), whereas multiple protons resulted in a more homogeneous line shape. Again, this irregular EPR line shape slightly distorted the simulated CHEESY profiles. Apart from these differences, the CHEESY profiles calculated with a different number of coupled protons are identical (Fig. [Fig App1.Ch1.S1.F28]c). This suggests that nuclear coupling does not affect the inversion profiles and can be neglected in the following considerations.

For these expanded spin systems, the relaxation superoperator ${\hat{\hat{R}}}_{2\rho }$ had to be adapted. Comparison of the performance of different relaxation matrices (data not shown) suggested that using a standard relaxation matrix for transversal relaxation where the diagonal elements $R_{ij,ij}$ are $T_{2\rho }^{-1}$ if |i〉⟶|j〉 is an electron spin transition is not sufficient to describe all relaxation pathways. Instead, $R_{ij,ij}=T_{2\rho }^{-1}$ was set for all elements i≠j with all other elements of ${\hat{\hat{R}}}_{2\rho }$ being zero. This is equivalent to defining an equal decoherence rate for all types of SL coherence. We assume that this becomes necessary as states in the tilted frame are mixed and the nuclear spin partially decoupled. A more thorough investigation of this effect is beyond the scope of this work and requires more analysis in the future.

### A15 Frame transformation of the Bloch equations

In order to perform calculations with the Bloch equations in the vector analogue of the tilted frame, we start with the Bloch equations in the rotating frame without relaxation terms [Bibr bib1.bibx6]:

A8
$${\dot{M}}^{\prime }=\left(\begin{array}{lll} 0 & -\Omega_{S} & \omega_{1}\\ \Omega_{S} & 0 & 0\\ -\omega_{1} & 0 & 0 \end{array}\right)M^{\prime }$$

where $M^{\prime }=(M_{x^{\prime }},M_{y^{\prime }},M_{z^{\prime }})$ is the magnetization vector and ${\dot{M}}^{\prime }$ its time derivative. We apply a rotation around $x^{\prime }$ , i.e., perpendicular to the MW field along $y^{\prime }$ , by $\theta ={\mathrm{Tan}}^{-1}(\omega_{1}/\Omega_{S})$ . In the following, we will use the following shorthand notation:

A9Rx(θ)=1000cos⁡(θ)-sin⁡(θ)0sin⁡(θ)cos⁡(θ)≡R,A10Rx(-θ)=Rx-1(θ)=1000cos⁡(θ)sin⁡(θ)0-sin⁡(θ)cos⁡(θ)≡R-1.

Multiplication from the left with R and insertion of $R^{-1}R=1$ yields the evolution of the magnetization vector in the tilted frame $M^{\prime \prime }=RM^{\prime }$ .

A11
$$\begin{array}{rl} {\dot{M}}^{\prime \prime } & =R\left(\begin{array}{lll} 0 & -\Omega_{S} & \omega_{1}\\ \Omega_{S} & 0 & 0\\ -\omega_{1} & 0 & 0 \end{array}\right)M^{\prime }\\ & =R\left(\begin{array}{lll} 0 & -\Omega_{S} & \omega_{1}\\ \Omega_{S} & 0 & 0\\ -\omega_{1} & 0 & 0 \end{array}\right)R^{-1}RM^{\prime }\\ & =R\left(\begin{array}{lll} 0 & -\Omega_{S} & \omega_{1}\\ \Omega_{S} & 0 & 0\\ -\omega_{1} & 0 & 0 \end{array}\right)R^{-1}M^{\prime \prime }. \end{array}$$

To obtain the new evolution matrix, we calculate the matrix product:

A12
$$\begin{array}{rl} & R\left(\begin{array}{lll} 0 & -\Omega_{S} & \omega_{1}\\ \Omega_{S} & 0 & 0\\ -\omega_{1} & 0 & 0 \end{array}\right)R^{-1}\\ & \;=\left(\begin{array}{lll} 1 & 0 & 0\\ 0 & \mathrm{cos}(\theta ) & -\mathrm{sin}(\theta )\\ 0 & \mathrm{sin}(\theta ) & \mathrm{cos}(\theta ) \end{array}\right)\left(\begin{array}{lll} 0 & -\Omega_{S} & \omega_{1}\\ \Omega_{S} & 0 & 0\\ -\omega_{1} & 0 & 0 \end{array}\right)\\ & \;\left(\begin{array}{lll} 1 & 0 & 0\\ 0 & \mathrm{cos}(\theta ) & \mathrm{sin}(\theta )\\ 0 & -\mathrm{sin}(\theta ) & \mathrm{cos}(\theta ) \end{array}\right)\\ & \;=\left(\begin{array}{lll} 1 & 0 & 0\\ 0 & \mathrm{cos}(\theta ) & -\mathrm{sin}(\theta )\\ 0 & \mathrm{sin}(\theta ) & \mathrm{cos}(\theta ) \end{array}\right)\\ & \;\left(\begin{array}{lll} 0 & -\Omega_{S}\mathrm{cos}(\theta )-\omega_{1}\mathrm{sin}(\theta ) & -\Omega_{S}\mathrm{sin}(\theta )+\omega_{1}\mathrm{cos}(\theta )\\ \Omega_{S} & 0 & 0\\ -\omega_{1} & 0 & 0 \end{array}\right)\\ & \;=\left(\begin{array}{lll} 0 & -\Omega_{S}\mathrm{cos}(\theta )-\omega_{1}\mathrm{sin}(\theta ) & -\Omega_{S}\mathrm{sin}(\theta )+\omega_{1}\mathrm{cos}(\theta )\\ \Omega_{S}\mathrm{cos}(\theta )+\omega_{1}\mathrm{sin}(\theta ) & 0 & 0\\ \Omega_{S}\mathrm{sin}(\theta )-\omega_{1}\mathrm{cos}(\theta ) & 0 & 0 \end{array}\right)\\ & \;=\left(\begin{array}{lll} 0 & -\omega_{\mathrm{eff}} & 0\\ \omega_{\mathrm{eff}} & 0 & 0\\ 0 & 0 & 0 \end{array}\right), \end{array}$$

where the last transformation step is performed with $\Omega_{S}=\omega_{\mathrm{eff}}\mathrm{cos}(\theta )$ and $\omega_{1}=\omega_{\mathrm{eff}}\mathrm{sin}(\theta )$ . Insertion into Eq. ([Disp-formula App1.Ch1.S1.E28]) yields the differential equations for the tilted-frame magnetization. In this tilted-frame expression, we add the transversal relaxation rates on the matrix diagonals, thus defining the decay of $M_{x^{\prime \prime }}$ and $M_{y^{\prime \prime }}$ .

A13
$$\begin{array}{rl} {\dot{M}}^{\prime \prime } & =\left(\begin{array}{lll} -1/T_{2\rho } & -\omega_{\mathrm{eff}} & 0\\ \omega_{\mathrm{eff}} & -1/T_{2\rho } & 0\\ 0 & 0 & 0 \end{array}\right)M^{\prime \prime }\\ & =\left(\begin{array}{l} -1/T_{2\rho }\cdot M_{x^{\prime \prime }}-\omega_{\mathrm{eff}}\cdot M_{y^{\prime \prime }}\\ \omega_{\mathrm{eff}}\cdot M_{x^{\prime \prime }}-1/T_{2\rho }\cdot M_{y^{\prime \prime }}\\ 0 \end{array}\right). \end{array}$$

This set of differential equations is solved by the standard approach of defining $M_{{+}^{\prime \prime }}=M_{x^{\prime \prime }}+iM_{y^{\prime \prime }}$ .

A14
$$\begin{array}{rl} {\dot{M}}_{{+}^{\prime \prime }} & ={\dot{M}}_{x^{\prime \prime }}+i{\dot{M}}_{y^{\prime \prime }}=\omega_{\mathrm{eff}}\left[-M_{y^{\prime \prime }}+iM_{x^{\prime \prime }}\right]\\ & -1/T_{2\rho }\cdot \left[M_{x^{\prime \prime }}+iM_{y^{\prime \prime }}\right]\\ & =\left[i\omega_{\mathrm{eff}}-1/T_{2\rho }\right]M_{{+}^{\prime \prime }} \end{array}$$

The solution of this differential equation is an exponential of the following form:

A15
$$\begin{array}{rl} M_{{+}^{\prime \prime }}(t) & =\left[M_{x^{\prime \prime }}(0)+iM_{y^{\prime \prime }}(0)\right]\\ & \cdot \left[\mathrm{cos}(\omega_{\mathrm{eff}}t)+i\mathrm{sin}(\omega_{\mathrm{eff}}t)\right]\cdot e^{-t/T_{2\rho }}. \end{array}$$

After factorization and separating the expression for $M_{{+}^{\prime \prime }}(t)$ into its real and imaginary parts, an expression for $M^{\prime \prime }(t)$ is obtained.

A16
$$M^{\prime \prime }(t)=\left(\begin{array}{l} \left[M_{x^{\prime \prime }}(0)\mathrm{cos}(\omega_{\mathrm{eff}}t)\right.\\ \left.\;\;\;-M_{y^{\prime \prime }}(0)\mathrm{sin}(\omega_{\mathrm{eff}}t)\right]\cdot e^{-t/T_{2\rho }}\\ \left[M_{y^{\prime \prime }}(0)\mathrm{cos}(\omega_{\mathrm{eff}}t)\right.\\ \left.\;\;\;+M_{x^{\prime \prime }}(0)\mathrm{sin}(\omega_{\mathrm{eff}}t)\right]\cdot e^{-t/T_{2\rho }}\\ M_{z^{\prime \prime }}(0) \end{array}\right)$$

The initial conditions in the tilted frame are calculated from the rotating-frame magnetization at t=0 , which is $M^{\prime }(0)=(0,0,M_{0})$ , so that, after back-transformation into the rotating frame, we end up with an analytical expression for $M^{\prime }(t)$ .

A17
$$\begin{array}{rl} & M^{\prime \prime }(0)=R\cdot M^{\prime }(0)=\left(\begin{array}{lll} 1 & 0 & 0\\ 0 & \mathrm{cos}(\theta ) & -\mathrm{sin}(\theta )\\ 0 & \mathrm{sin}(\theta ) & \mathrm{cos}(\theta ) \end{array}\right)\left(\begin{array}{l} 0\\ 0\\ M_{0} \end{array}\right)\\ & \;=\left(\begin{array}{l} 0\\ -\mathrm{sin}(\theta )M_{0}\\ \mathrm{cos}(\theta )M_{0} \end{array}\right),\\ & M^{\prime \prime }(t)=\left(\begin{array}{l} \mathrm{sin}(\omega_{\mathrm{eff}}t)\mathrm{sin}(\theta )M_{0}\cdot e^{-t/T_{2\rho }}\\ -\mathrm{cos}(\omega_{\mathrm{eff}}t)\mathrm{sin}(\theta )M_{0}\cdot e^{-t/T_{2\rho }}\\ \mathrm{cos}(\theta )M_{0} \end{array}\right),\\ & M^{\prime }(t)=R^{-1}M^{\prime \prime }(t)=\left(\begin{array}{lll} 1 & 0 & 0\\ 0 & \mathrm{cos}(\theta ) & \mathrm{sin}(\theta )\\ 0 & -\mathrm{sin}(\theta ) & \mathrm{cos}(\theta ) \end{array}\right)\\ & \;\left(\begin{array}{l} \mathrm{sin}(\omega_{\mathrm{eff}}t)\mathrm{sin}(\theta )M_{0}\cdot e^{-t/T_{2\rho }}\\ -\mathrm{cos}(\omega_{\mathrm{eff}}t)\mathrm{sin}(\theta )M_{0}\cdot e^{-t/T_{2\rho }}\\ \mathrm{cos}(\theta )M_{0} \end{array}\right)\\ & =\left(\begin{array}{l} \mathrm{sin}(\omega_{\mathrm{eff}}t)\mathrm{sin}(\theta )M_{0}\cdot e^{-t/T_{2\rho }}\\ \left[-\mathrm{cos}(\omega_{\mathrm{eff}}t)\mathrm{sin}(\theta )\mathrm{cos}(\theta )\cdot e^{-t/T_{2\rho }}+\mathrm{sin}(\theta )\mathrm{cos}(\theta )\right]M_{0}\\ \left[\mathrm{cos}(\omega_{\mathrm{eff}}t){\mathrm{sin}}^{2}(\theta )\cdot e^{-t/T_{2\rho }}+{\mathrm{cos}}^{2}(\theta )\right]M_{0} \end{array}\right). \end{array}$$

In the limit of long evolution times, all terms with $e^{-t/T_{2\rho }}$ decay to zero, and a reduced magnetization vector remains:

A18
$$\begin{array}{rl} M(t⟶\infty ) & =\underset{t⟶\infty }{lim}\left(\begin{array}{l} \mathrm{sin}\left(\omega_{\mathrm{eff}}t\right)\mathrm{sin}(\theta )M_{0}\cdot e^{-t/T_{2\rho }}\\ \left[-\mathrm{cos}\left(\omega_{\mathrm{eff}}t\right)\mathrm{sin}(\theta )\mathrm{cos}(\theta )\right.\\ \left.\;\;\;\cdot e^{-t/T_{2\rho }}+\mathrm{sin}(\theta )\mathrm{cos}(\theta )\right]M_{0}\\ \left[+\mathrm{cos}\left(\omega_{\mathrm{eff}}t\right){\mathrm{sin}}^{2}(\theta )\right.\\ \left.\;\;\;\cdot e^{-t/T_{2\rho }}+{\mathrm{cos}}^{2}(\theta )\right]M_{0} \end{array}\right)\\ & =\left(\begin{array}{l} 0\\ \mathrm{sin}(\theta )\mathrm{cos}(\theta )M_{0}\\ {\mathrm{cos}}^{2}(\theta )M_{0} \end{array}\right)\\ & =\left(\begin{array}{l} 0\\ \frac{\left(\nu -\nu_{\mathrm{off}}\right)\nu_{1}}{\nu_{1}^{2}+{\left(\nu -\nu_{\mathrm{off}}\right)}^{2}}\\ 1-\frac{\nu_{1}^{2}}{\nu_{1}^{2}+{\left(\nu -\nu_{\mathrm{off}}\right)}^{2}} \end{array}\right), \end{array}$$

where the last step of the rearrangement was done using Mathematica.

### A16 Solution of Bloch equations in the rotating frame

When adding the transversal relaxation terms in the rotating frame, Eq. ([Disp-formula App1.Ch1.S1.E36]) is obtained [Bibr bib1.bibx6]:

A19
$$\begin{array}{rl} {\dot{M}}^{\prime } & =\left(\begin{array}{l} {\dot{M}}_{x^{\prime }}\\ {\dot{M}}_{y^{\prime }}\\ {\dot{M}}_{z^{\prime }} \end{array}\right)\\ & =\left(\begin{array}{lll} -1/T_{2} & -\Omega_{S} & \omega_{1}\\ \Omega_{S} & -1/T_{2} & 0\\ -\omega_{1} & 0 & 0 \end{array}\right)\left(\begin{array}{l} M_{x^{\prime }}\\ M_{y^{\prime }}\\ M_{z^{\prime }} \end{array}\right). \end{array}$$

As the analytical solution of Eq. ([Disp-formula App1.Ch1.S1.E36]) is rather cumbersome and provides little intuitive insight into the physical processes [Bibr bib1.bibx62], we used a numerical differential equation solver implemented in MATLAB (ode45) to obtain the results displayed in Fig. [Fig Ch1.F8].

## Appendix B Additional information on the figures in the main text

In the following, the experimental conditions and data processing steps of the figures in the main text are given. This includes a reference to the experimental data sets and processing scripts that are uploaded in a separate data repository (10.25625/B11CUC, [Bibr bib1.bibx43]). The experimental data sets are provided as .DTA and .DSC files and were processed with the eprload command implemented in EasySpin [Bibr bib1.bibx59]. The processing and simulation files are provided as MATLAB code. Additionally, the analysis files for the figures in Appendix A can be found in the data repository.

### B1 Fig. 2 (SL Rabi nutations)

SL Rabi nutations obtained with the sequence in Fig. [Fig Ch1.F2]a had the following parameters: T=100K , $t_{\pi /2}=18\;\mathrm{ns}$ , $t_{\pi }=36\;\mathrm{ns}$ , τ=1µs , $\tau_{0}=1\;µs$ , $\tau_{1}=2\;µs$ , $a_{\mathrm{PM}}=0.4$ , shot repetition time (SRT) =40ms , shots per point (SPP) = 4, and scans = 3. A four-step phase cycle of the π refocusing pulse was used:

$$\phi_{\pi }=[0{}^{\circ},90{}^{\circ},180{}^{\circ},270{}^{\circ}],\;\phi_{\det}=[+,-,+,-].$$

Rabi traces were obtained by recording the integrated echo intensity as a function of $t_{\mathrm{PM}}$ . For the Fourier transformation, the traces were corrected for phase and baseline and apodized with a half-cosine bell function. This apodized data set was filled with zeros with twice the number of data points and Fourier transformed using the fft command in MATLAB. The simulation procedure is illustrated in Fig. [Fig Ch1.F2]c. The same overall trace length and FT procedure as in the experiment was used for the simulations. Experimental data sets: 999a–999f. Processing file: SL_Rabi_nutations_analysis.m.

### B2 Fig. 3 (SL echoes and WOP SL echoes)

SL echoes and WOP SL echoes obtained with the sequences in Fig. [Fig Ch1.F3]a and b, respectively, used the following parameters: T=100K , $t_{\pi /2}=18\;\mathrm{ns}$ , $t_{\pi }=36\;\mathrm{ns}$ , τ=0.3µs , $\tau_{0}=2\;µs$ , $\tau_{1}=3\;µs$ , $\tau_{3}=10.7\;µs-\tau_{2}$ , $a_{\mathrm{PM}}=0.4$ , $\nu_{\mathrm{PM}}=15\;\mathrm{MHz}$ , $t_{\mathrm{PM}1}=t_{\mathrm{PM}3}=80\;\mathrm{ns}$ , $t_{\mathrm{PM}2}=160\;\mathrm{ns}$ , SRT =40ms , SPP = 1–4, and scans = 5. In addition to the PCs denoted in the figure, a two-step PC of PM3 was performed in all cases:

$$\phi_{\mathrm{PM}3}=[0{}^{\circ},180{}^{\circ}],\;\phi_{\det}=[+,-].$$

Echoes were obtained by recording the integrated echo intensity as a function of $\tau_{2}$ . The echo traces were phase corrected so that the dominant signal in the trace without PC of PM2 was maximized in the real part of the signal. The experiments with different PCs were subsequently corrected with the same phase factor. Experimental data sets: 967a–967f. Processing files: SL_echo_analysis.m and WOP_SL_echo_analysis.m.

### B3 Fig. 4 (SL relaxation measurements)

SL echo decay traces were obtained using the sequence in Fig. [Fig Ch1.F3]a. The following parameters were used for the trace in Fig. [Fig Ch1.F4]a: T=100K , $t_{\pi /2}=20\;\mathrm{ns}$ , $t_{\pi }=40\;\mathrm{ns}$ , τ=0.3µs , $\tau_{0}=100\;µs$ , $\tau_{1}=\tau_{2}$ , $\tau_{3}=32.2\;µs-\tau_{1}-\tau_{2}$ , $a_{\mathrm{PM}}=0.4$ , $\nu_{\mathrm{PM}}=15\;\mathrm{MHz}$ , $t_{\mathrm{PM}1}=t_{\mathrm{PM}3}=80\;\mathrm{ns}$ , $t_{\mathrm{PM}2}=160\;\mathrm{ns}$ , SRT = 40ms , SPP = 4, and scans = 3. The trace was obtained by recording the integrated echo intensity as a function of $\tau_{1}=\tau_{2}$ . A two-step PC of PM3 was performed. The bare-state echo decay was obtained using the Hahn echo sequence ( π/2-τ-π-τ-echo ) with $t_{\pi /2}=22\;\mathrm{ns}$ and $t_{\pi }=44\;\mathrm{ns}$ . The experiments in (b) were performed with similar parameters, but $t_{\mathrm{PM}}$ was, for each experiment, adjusted to the different SL Rabi frequencies. Hahn echoes with lower $\nu_{1}$ were detected with a 50/100ns and 200/400ns echo. Carr–Purcell experiments were performed with $t_{\pi /2}=22\;\mathrm{ns}$ , and $t_{\pi }=44\;\mathrm{ns}$ . A 16-step PC of the last two π pulses was applied:

$$\begin{array}{c} \phi_{\pi ,N}=[0{}^{\circ}{]}_{4},[90{}^{\circ}{]}_{4},[180{}^{\circ}{]}_{4},[270{}^{\circ}{]}_{4},\\ \phi_{\pi ,N+1}=[0{}^{\circ},90{}^{\circ},180{}^{\circ},270{}^{\circ}{]}_{4},\\ \phi_{\det}=[+,-,+,-,-,+,-,+{]}_{2}. \end{array}$$

Experimental data sets: 1018 , 1131 , and 1134 . Processing file: T2rho_analysis.m.

### B4 Figs. 5 and 6 (CHEESY $z^{\prime }$ profiles)

CHEESY $z^{\prime }$ profiles were measured using the sequence in Fig. [Fig Ch1.F5]a and the following parameters: T=100K , $t_{\pi /2,\mathrm{chirp}}=1\;µs$ , $t_{\pi ,\mathrm{chirp}}=0.5\;µs$ , τ=3µs , SRT =40ms , SPP = 150, and scans = 1. An eight-step PC of the chirp echo was used:

$$\begin{array}{c} \phi_{\pi /2,\mathrm{chirp}}=[0{}^{\circ}{]}_{4},\;[180{}^{\circ}{]}_{4},\\ \phi_{\pi ,\mathrm{chirp}}=[0{}^{\circ},90{}^{\circ},180{}^{\circ},270{}^{\circ}{]}_{2},\\ \phi_{\det}=[+,-{]}_{2},\;[-,+{]}_{2}. \end{array}$$

The chirp pulses had a sweep width of 200MHz centered at the maximum of the resonator dip. The frequency sweep of the chirp pulses was adjusted to compensate for the resonator profile ( FWHM≈140MHz ), yielding an approximately constant adiabaticity [Bibr bib1.bibx17]. A wideband, uniform rate, smooth truncation (WURST) amplitude modulation [Bibr bib1.bibx39] was applied to yield the final chirp pulse.

Before the experiment, the magnetic field was adjusted so that the BDPA EPR line was centered at $\nu_{\mathrm{off}}=-31\;\mathrm{MHz}$ , and the analyzed pulse was applied with the same frequency offset. FT was performed using the fft command in MATLAB after choosing a FT window of 5µs symmetrically around the echo, apodization with a cosine bell function and zero filling with the number of zeros equal to the length of the data set. Zeroth-order phase correction was performed so that the integral of the FT was maximized in the real part of the signal. Experimental data set: 1025 . Processing files: CHEESY_z_profile_analysis.m and CHEESY_z_profile_SimSpinach.m.

### B5 Fig. 7 (CHEESY $x^{\prime }y^{\prime }$ profiles)

The CHEESY $x^{\prime }y^{\prime }$ profiles in Fig. [Fig Ch1.F7]c and d were obtained using the pulse sequence in (a) and T=100K . The chirp pulses were identical to the π chirp pulse in the previous section. The delays were set to $\tau_{1}=1\;µs$ and $\tau_{2}=4\;µs$ , together with SRT =40ms , SPP = 250, scans = 1, and an offset $\nu_{\mathrm{off}}=-29\;\mathrm{MHz}$ . A 32-step PC was used:

$$\begin{array}{c} \phi_{\mathrm{analyzed}}=[0{}^{\circ}{]}_{16},[180{}^{\circ}{]}_{16};\\ \phi_{\pi ,\mathrm{chirp}(1)}=\{[0{}^{\circ}{]}_{4},[90{}^{\circ}{]}_{4},[180{}^{\circ}{]}_{4},[270{}^{\circ}{]}_{4}{\}}_{2};\\ \phi_{\pi ,\mathrm{chirp}(2)}=[0{}^{\circ},90{}^{\circ},180{}^{\circ},270{}^{\circ}{]}_{8}:\\ \phi_{\det}=\{[+,-{]}_{2},[-,+{]}_{2}{\}}_{2},\{[-,+{]}_{2},[+,-{]}_{2}{\}}_{2}) \end{array}$$

After manual selection of an FT window with 4µs length, FT was performed as in the previous section. Zeroth-order phase correction was performed so that the dispersive signal was maximized in the imaginary channel.

The measurement of $T_{1\rho }$ (e) was performed with the sequence in (b), where $t_{\pi /2}=14\;\mathrm{ns}$ , $t_{\pi }=28\;\mathrm{ns}$ , τ=1µs , and $\nu_{1,\mathrm{SL}}=0.8\;\mathrm{MHz}$ . The intensity was scaled to overlap with the data points from the R-CHEESY experiment. Experimental data sets: 875 , 876 , and 968 . Processing file: CHEESY_xy_profile_analysis.m.

### B6 Fig. 8 (Bloch equations)

The experimental data shown in (a) and (b) are the same as in Figs. [Fig Ch1.F5]c and [Fig Ch1.F7]c. Processing files: CHEESY_z_profile_analysis.m, CHEESY_xy_profile_analysis.m., and Bloch_simulations.m
